# Computationally Assisted Structural Elucidation of Cembranoids from the Soft Coral *Sarcophyton tortuosum*

**DOI:** 10.3390/md20050297

**Published:** 2022-04-27

**Authors:** Chih-Hua Chao, Kuan-Hua Lin, Chiung-Yao Huang, Tsong-Long Hwang, Chang-Feng Dai, Hui-Chi Huang, Jyh-Horng Sheu

**Affiliations:** 1 School of Pharmacy, China Medical University, Taichung 406040, Taiwan; chchao@mail.cmu.edu.tw; 2 Chinese Medicine Research and Development Center, China Medical University Hospital, Taichung 404332, Taiwan; 3 Department of Marine Biotechnology and Resources, National Sun Yat-sen University, Kaohsiung 80424, Taiwan; m005020002@student.nsysu.edu.tw (K.-H.L.); betty8575@yahoo.com.tw (C.-Y.H.); 4 Graduate Institute of Natural Products, College of Medicine, Chang Gung University, Taoyuan 33303, Taiwan; htl@mail.cgu.edu.tw; 5 Research Center for Chinese Herbal Medicine, College of Human Ecology, Chang Gung University of Science and Technology, Taoyuan 33303, Taiwan; 6 Research Center for Food and Cosmetic Safety, College of Human Ecology, Chang Gung University of Science and Technology, Taoyuan 33303, Taiwan; 7 Graduate Institute of Health Industry Technology, College of Human Ecology, Chang Gung University of Science and Technology, Taoyuan 33303, Taiwan; 8 Department of Anesthesiology, Chang Gung Memorial Hospital, Taoyuan 33303, Taiwan; 9 Institute of Oceanography, National Taiwan University, Taipei 10617, Taiwan; corallab@ntu.edu.tw; 10 Department of Chinese Pharmaceutical Sciences and Chinese Medicine Resources, China Medical University, Taichung 40402, Taiwan; hchuang@mail.cmu.edu.tw; 11 Department of Medical Research, China Medical University Hospital, China Medical University, Taichung 404332, Taiwan; 12 Graduate Institute of Natural Products, Kaohsiung Medical University, Kaohsiung 80708, Taiwan

**Keywords:** tortuolide A, tortuolide B, *epi*-sarcophytonolide, DP4+, *J*-DP4, *Sarcophyton tortuosum*

## Abstract

A persistent study on soft coral *Sarcophyton tortuosum* resulted in the characterization of two new cembranolides, tortuolides A and B (**1** and **2**), and a new related diterpene, *epi*-sarcophytonolide Q. Their structures were determined not only by extensive spectroscopic analysis but also by DFT calculations of ECD and NMR data, the latter of which was combined with statistical analysis methods, e.g., DP4+ and *J*-DP4 approaches. Anti-inflammatory and cytotoxicity activities were evaluated in this study.

## 1. Introduction

Soft corals are known to produce a large variety of secondary metabolites [1]. In particular, soft coral of the genus *Sarcophyton* is a prolific source of promising bioactive cembranoids, some of which have exhibited antiviral [2], anti-inflammatory [3,4,5,6,7], and cytotoxic activities [4,5,6,8]. The flexibility of the macrocyclic ring in cembranoids makes the accurate determination of chemical structures particularly challenging. Despite the 2D NMR spectroscopic method being the most potent approach for structural elucidation, it suffers from inherently low accuracy for flexible structures, especially when there is no informative correlation in NMR spectrometry. Consequently, the computational approach and related statistical analysis methods, e.g., DP4+ and *J*-DP4 [9,10], have gradually became convenient and reliable alternatives.

**Figure 1:**
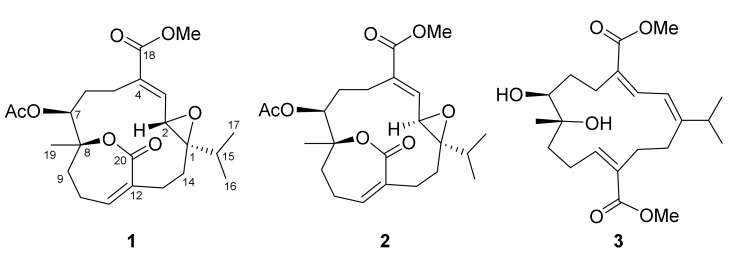
Structures of compounds **1**–**3**.

Our pervious investigation on *Sarcophyton tortuosum* resulted in the isolation of several novel structures, including secotortuosenes A and B with a novel secoditerpenoidal skeleton, bistortuolide cyclobutane A with a novel cyclobutane biscembranoidal skeleton, and tortuosenes A and B with a rare tricyclic diterpenoidal skeleton [7,11]. As part of our continuing effort to explore bioactive marine natural products from soft corals [3,4,5,6,7,8], the chemical constituents of *S. tortuosum* collected at Lanyu Island were investigated in this study, and three new cembranoids, namely tortuolides A and B (**1** and **2**) and *epi*-sarcophytonolide Q (**3**), were characterized (Figure 1). Structural elucidations were performed by a comprehensive 2D NMR spectroscopic analysis, as well as computational and statistical analysis methods. Their biological activities, including cytotoxicity and anti-inflammatory activities, were evaluated herein.

## 2. Results and Discussion

Defrosted specimens (1.3 kg) of the soft coral *S.* *tortuosum* was freeze-dried, minced, and extracted with EtOAc to yield a crude extract (10.2 g), which was repeatedly chromatographed on silica gel and subsequently purified by high-performance liquid chromatography (HPLC) to obtain compounds **1** (8.9 mg), **2** (2.7 mg), and **3** (2.1 mg) (Figure 1).

**Figure 2:**
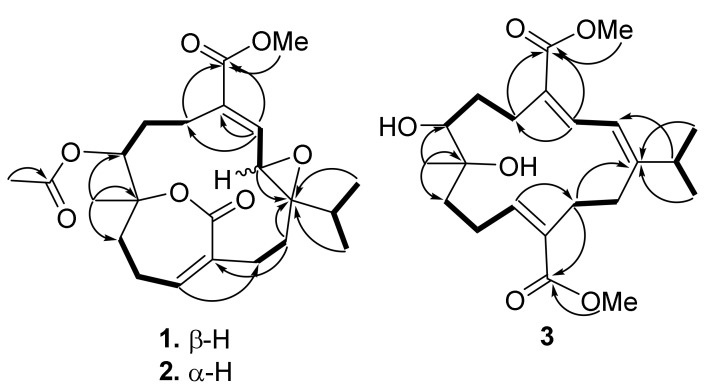
COSY (bold) and selective HMBC (arrows) correlations of **1**–**3**.

**Figure 3:**
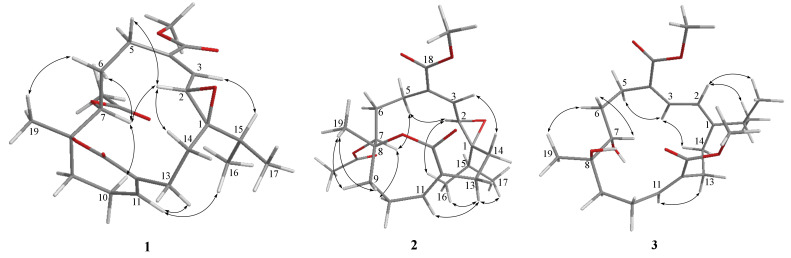
Selective NOE correlations of **1**–**3**.

Tortuolide A (**1**), obtained as a colorless oil, was found to have a molecular formula of C_23_H_32_O_7_ based on the sodiated ion peak at *m*/*z* 443.2049 [M + Na]^+^ (calcd for C_23_H_32_O_7_Na, 443.2040). The ^1^H and ^13^C NMR data (Table 1) revealed evidence of two α,β-conjugated carboxylate systems [*δ*_C_ 136.9 (CH, C-3), 136.3 (C, C-4), and 167.0 (C, C-18); *δ*_H_ 6.69 (1H, d, *J* = 10.0 Hz, H-3); *δ*_C_ 140.4 (CH, C-11), 132.2 (C, C-12), and 167.6 (C, C-20); *δ*_H_ 6.33 (1H, br s, H-11)], an acetoxyl group [ *δ*_C_ 169.4 (C), 21.1 (CH_3_); *δ*_H_ 1.99 (s, CH_3_)], an epoxy group [*δ*_C_ 68.4 (C, C-1), 59.2 (CH, C-2); *δ*_H_ 3.28 (1H, d, *J* = 10.0 Hz, H-2)], an oxygenated methine [*δ*_C_ 69.8 (CH, C-7); *δ*_H_ 4.93 (1H, dd, *J* = 8.5, 2.0 Hz, H-7)], a methoxyl group [*δ*_C_ 52.0 (CH_3_, 18-OMe); *δ*_H_ 3.73 (3H, s, 18-OMe)], and an oxygenated quaternary carbon [*δ*_C_ 83.5 (C, C-8)]. Inspection of the NMR data revealed that the planar structures of **1** and emblide [11,12] were quite similar, with differences for the Δ^1^ double bond in emblide replaced by an epoxy ring in **1**, as indicated by the heteronuclear multiple bond correlation (HMBC) correlations from H_3_-16, H_3_-17, H_2_-14, and H-2 to C-1, as well as the correlation spectroscopy (COSY) correlation between H-2 and H-3 (Figure 2). The cis geometry of the epoxide was assigned by the nuclear Overhauser effect (NOE) correlation of H-15/H-3, whereas the *E* double bond was deduced based on the NOE correlation of OMe/H-3 (Figure 3). The correlation of H-7/H-2 suggested both protons were pointed inside the macrocyclic ring. Furthermore, NOE correlations of H_3_-19/H-6 (*δ*_H_ 2.37), H-7/H-10 (*δ*_H_ 2.28), and H-11/H_3_-16 suggested H_3_-19 and H-7 were oppositely oriented (Supplementary Materials, Figures S1–S8).

**Figure 4:**
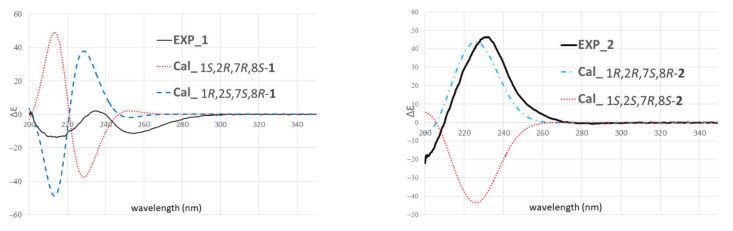
Experimental and calculated ECD spectra of (**1**) and (**2**). Gaussian band shape (σ) with values of 0.20 (for **1**) and 0.28 (for **2**) eV.

The relative configuration of **1** was further secured by utilizing the computational NMR data coupled with a combined indirect *J*-DP4 (i*J*-DP4) and direct *J*-DP4 (d*J*-DP4) [10]. Four possible diastereomers, including 1R*,2S*,7S*,8S*-**1**, 1R*,2S*,7R*,8S*-**1**, 1R*,2S*,7S*,8R*-**1**, and 1R*,2S*,7R*,8R*-**1**, were subjected to conformational search using the Merck molecular force field (MMFF94) as implemented in the GMMX program. In this case, ^3^*J*_H,H_ values of H-2/H-3 and H-7/H_2_-6 were selected to restrict conformational sampling (Supplementary Materials, Tables S3–S6). Moreover, in order to reduce the computational cost, the strong NOE correlation of H_3_-16/H-11 in compound **1** (Figure 3) was further selected as second restriction. After restrictions by ^3^*J*_H,H_ values and NOE correlations, two of the diastereomers, 1R*,2S*,7S*,8S*-**1** and 1R*,2S*,7R*,8S*-**1**, where the H_3_-16 and H-11 are anti-oriented, were excluded, as this correlation (H_3_-16/H-11) is not possible in these two candidates. On the other hand, the remaining two diastereomers without geometry optimization, 1R*,2S*,7S*,8R*-**1** and 1R*,2S*,7R*,8R*-**1**, were subjected to gauge-invariant atomic orbital (GIAO) calculations of shielding tensors and coupling constants. The Boltzmann weighted computational data were analyzed utilizing the *J*-DP4 statistical method [10]. As a result, 1R*,2S*,7S*,8R*-**1** was found to be the correct structure, with a high probability of 99.47% (Table 2). The absolute configuration of **1** was further determined by comparing the experimental and calculated electronic circular dichroism (ECD) spectra (Supplementary Materials, Table S1). The calculated ECD spectra (Figure 4) at the TDDFT/M06-2X/def2tzvp level of theory with integral equation formalism polarizable continuum model (IEFPCM) in MeOH suggested the absolute configuration of **1** to be 1*R*, 2*S,* 7*S*, and 8*R*.

Tortuolide B (**2**) was found to have the same molecular formula (C_23_H_32_O_7_) as that of **1**, with the sodiated ion peak at *m*/*z* 443.2044 [M + Na]^+^ (calcd for C_23_H_32_O_7_Na, 443.2040). After careful analysis of the differences between compounds **1** and **2**, it was found that the two compounds shared the same planar structure, and **2** should be a configurational isomer of **1**. The HMBC and COSY correlations confirmed the above elucidation as shown in Figure 2. The NOE correlations of H-3/H-14 (*δ*_H_ 1.90) and H-2/H-5 (*δ*_H_ 2.71) suggested the geometries of trans epoxide and *E*-double bond, respectively (Figure 3). The NOE correlations of H-5 (*δ*_H_ 2.71)/H-2 and H-5/H-7 indicated these protons were α-oriented. The H-11 in unsaturated ε-lactone was assigned to a point on the same face with the isopropyl group due to the observation of NOE correlations of H-13 (*δ*_H_ 2.20)/H-11, H-13/H_3_-16, and H-13/H_3_-17. Moreover, correlations of H_2_-9/H_3_-19 and H-7/H-9 (*δ*_H_ 2.11) indicated H_3_-19 located opposite to H-7 (Supplementary Materials, Figures S9–S16). Similarly, the relative configurations were also confirmed by computational NMR calculation for the four possible diastereomers (1R*,2R*,7R*,8R*-**2**, 1R*,2R*,7S*,8R*-**2**, 1R*,2R*,7R*,8S*-**2**, and 1R*,2R*,7S*,8S*-**2**) and combined with subsequent *J*-DP4 analysis. The diastereomer with 1R*,2R*,7S*,8S* configuration was excluded using ^3^*J*_H,H_ values (^3^*J*_H,H_ values of H-2/H-3 and H-7/H_2_-6) as conformational constrains (Supplementary Materials, Tables S7–S10). As a result, 1R*,2R*,7S*,8R*-**2** was suggested to be the correct structure, with a high *J*-DP4 probability of 100% (Table 3). Furthermore, the theoretical ECD spectra were calculated for 1*R*,2*R*,7*S*,8*R*-**2** and its enantiomer, 1*S*,2*S*,7*R*,8*S*-**2** (Supplementary Materials, Table S2), and the former showed good agreement with the calculated data (Figure 4).

*epi*-Sarcophytonolide Q (**3**), a white amorphous powder, was found to have a molecular formula of C_22_H_34_O_6_, as determined by HRESIMS (*m*/*z* calcd 417.2248; found 417.2251, [M + Na]^+^). The IR spectrum of **3** showed the presence of hydroxyl (*ν*_max_ 3474 cm^−1^) and a conjugated carbonyl group (*ν*_max_ 1699 cm^−1^). The latter was confirmed by the UV absorption maxima (λ_max_ 286, 216). The NMR spectroscopic data of **3** revealed the presence of two conjugated methyl esters [*δ*_C_ 168.0 (C, C-18), 51.2 (CH_3_, 18-OMe); *δ*_H_ 3.75 s (18-OMe), and *δ*_C_ 168.5 (C, C-20), 51.6 (CH_3_, 20-OMe); *δ*_H_ 3.62 s (20-OMe)], an oxymethine [*δ*_C_ 70.3 (CH); *δ*_H_ 3.70 d (*J* = 10.4 Hz, H-7)], and an oxygenated quaternary carbon [*δ*_C_ 74.6 (C, C-8)] (Table 1 and Table 2). The above data were very similar to those of sarcophytonolide Q [13]. Further COSY and HMBC correlations confirmed that they share the same planar structure (Figure 2). In the NOESY spectra of **3**, H_3_-19 showed an NOE correlation with H-6 (*δ*_H_ 1.53) but not with H-7, which was reported in sarcophytonolide Q, revealing they were diastereomers (Supplementary Materials, Figures S17–S24). The above data inferred that they are structurally different at either C-7 or C-8; however, both compounds shared the same coupling constant at H-7 (10.4 Hz), revealing that the constraints for the *J*-DP4 method were unable to be applied in this case. Alternatively, the DP4+ method was performed for two possible diastereomers of **3** (7S*,8R*-3 and 7R*,8R*-3) [9]. First, a conformational search was performed using GMMX, with subsequent geometry optimization at the B3LYP/6-31G(d) level of theory. Next, the NMR shielding tensors were calculated at the mPW1PW91/6-31+G(d,p) level with the polarizable continuum model (PCM) in CHCl_3_, as recommended in the literature [9]. Finally, the Boltzmann-weighted data were subjected to DP4+ analysis. As a result, a relative 7S*,8R* configuration was suggested for **3** with 100% probability (Table 3).

Evaluations for inhibitory effect toward the superoxide anion generation and elastase release in fMLF/CB-induced human neutrophils were performed on compounds **1**–**3**. The result showed that compounds **1** and **2** exhibited weak inhibitory activity of 13.64 ± 2.27 and 14.15 ± 3.57%, respectively, on elastase release at a concentration of 10 μM. Compounds **1**–**3** were further screened for cytotoxicity toward murine leukemia (P388), human chronic myelogenous leukemia (K562), human colon carcinoma (HT-29), human lung adenocarcinoma (A-549), and lymphoblastic leukemia (Molt-4); unfortunately, the tested compounds were also found to be inactive against the above cell lines, with IC_50s_ over 40 μM. 

## 3. Materials and Methods

### 3.1. General Experimental Procedures

Specific optical rotations were measured in CHCl_3_ using a JASCO P-1020 digital polarimeter (JASCO Corporation, Tokyo, Japan). IR spectra were recorded on a JASCO FT/IR-4100 spectrometer and an FT/IR-4100 infrared spectrophotometer (JASCO Corporation, Tokyo, Japan). The NMR experiments were performed in CDCl_3_ on a Varian 400MR FT-NMR instrument and a Varian Unity INOVA 500 FT-NMR spectrometer (Varian Inc., Palo Alto, CA, USA). LR- and HR-ESIMS were measured with a Bruker APEX II mass spectrometer and a Bruker Apex-Qe 9.4T mass spectrometer (Bruker, Bremen, Germany), respectively. Before column chromatography using Si gel or C18 gel (40–63 µm, Merck, Darmstadt, Germany), TLC analysis was performed on aluminum plates coated with Si gel or C18 gel (Kieselgel 60 F_254_, 0.25 mm, Merck, Darmstadt, Germany). HPLC was performed on a Hitachi L-2455 apparatus equipped with a Supelco C18 column (ODS-3, 5 μm, 250 × 20 mm; Sciences Inc., Tokyo, Japan).

### 3.2. Animal Material

The animal material, *S. tortuosum*, was manually collected by an underwater diver from the coral reef of Lanyu Island of Taiwan in August 2008. The specimen was identified by Prof. C.-F. Dai. A voucher specimen (specimen no. sheuCYJ-001) was deposited with the Department of Marine Biotechnology and Resources, National Sun Yat-sen University, Kaohsiung 804, Taiwan.

### 3.3. Extraction and Separation

The defrosted *S. tortuosum* organism was weighed and subsequently freeze-dried, minced, and extracted repeatedly (1L) with ethyl acetate (EtOAc) to obtain a crude product (10.2 g), which was fractionated to obtain 25 fractions (F1-F25) as described previously [11]. Fractions 18 and 19, showing similar compositions on TLC plates, were combined and fractionated by chromatography on Si gel using *n*-hexane-EtOAc (3:1) and then *n*-hexane-acetone (6:1 and 3:1) as eluents to yield a crude residue, which was purified by normal-phase HPLC (*n*-hexane-EtOAc, 5:2) to give compound **3** (2.1 mg).

A subfraction purified by an Si gel open column (*n*-hexanes-EtOAc, 5:1) from fraction 16 was further separated by semipreparative NP-HPLC eluting with *n*-hexane-EtOAc (5:1) to afford a crude residue, which was further purified by reverse-phase HPLC (CH_3_CN-H_2_O, 3:2) to give compounds **1** (8.9 mg) and **2** (2.7 mg).

Tortuolide A (**1**): colorless oil; [α]^26^_D_ + 24 (c 0.74, CHCl_3_); UV (MeOH) λ_max_ (log ε) 282 (3.2), 228 (4.0) nm; IR (KBr) ν_max_ 2961, 2927, 1740, 1713, 1693, 1260, 1212 cm^−1^; ^1^H and ^13^C NMR data, see Table 1; positive ESIMS *m*/*z* 443 [M + Na]^+^; positive HRESIMS *m*/*z* 443.2049 [M + Na]^+^ (calcd for 443.2040 for C_23_H_32_O_7_Na).

Tortuolide B (**2**): colorless oil; [α]^26^_D_ +111 (c 0.77, CHCl_3_); UV (MeOH) λ_max_ (log ε) 228 (4.2) nm; IR (KBr) ν_max_ 2928, 2857, 1719, 1704, 1253, 1208 cm^−1^; ^1^H and ^13^C NMR data, see Table 1; positive ESIMS *m*/*z* 443 [M + Na]^+^; positive HRESIMS *m*/*z* 443.2044 [M + Na]^+^ (calcd for 443.2040 for C_23_H_32_O_7_Na).

*epi*-Sarcophytonolide Q (**3**): white amorphous solid; [α]^25^_D_ +214 (c 1.43, CHCl_3_); UV (MeOH) λ_max_ (log ε) 286 (4.1), 216 (4.0) nm; IR (KBr) ν_max_ 3474, 3016, 2955, 2873, 1698, 1629, 1439, 1378 cm^−1^; ^1^H and ^13^C NMR data, see Table 1; positive ESIMS *m*/*z* 417 [M + Na]^+^; positive HRESIMS *m*/*z* 417.2251 [M + Na]^+^ (calcd for 417.2248 for C_22_H_34_O_6_Na).

### 3.4. Computational Method

A conformational search was performed at the MMFF94 force field using GMMX package implemented in Gaussian 16 software [14]. The resulted conformers, within a 6 kcal/mol window, were subjected for further NMR and ECD calculations.

For i*J*/d*J*-DP4, the shielding tensors and Fermi contacts were calculated at the PCM/ B3LYP/6-31+G(d,p)//MMFF94 level. The resulting data were weighted based on Boltzmann population using energies calculated at the same level of theory. *J*-DP4 probabilities were generated using the Excel sheet provided by Zanardi et al [10]. For DP4+, the conformers were subjected to geometry optimization at B3LYP/6-31G(d) [9]. The NMR chemical shifts were computed at the PCM/mPW1PW91/6-31G+(d,p)//B3LYP/6-31G(d) level in chloroform with the Boltzmann population refined in the solvation model based on density (SMD) for CHCl_3_ at a new level (M06-2X/6-31G+(d,p)) [15]. The DP4+ probability was determined using the Excel sheet provided in the literature [9]. 

The conformers resulting from MMFF94 calculations were subjected to geometry optimizations and frequency calculations at the M06-2X/def2svp level using IEFPCM in MeOH. The generated ECD spectra calculated at TDDFT/M06-2X/def2TZVP with IEFPCM in MeOH were weighted based on the Boltzmann population using Gibbs free energy, obtained by the sum of single-point energy at M06-2X/def2TZVP and the thermal correction at M06-2X/def2svp. The calculated ECD spectra were generated using GaussView 6 by applying a Gaussian band shape with 0.20 and 0.28 eV width for **1** and **2**, respectively. It should be noted that Grimme’s dispersion (D3 version) was used for empirical dispersion correction in ECD calculation, and the *g09defaults* keywords were applied for NMR calculation.

### 3.5. Cytotoxicity Assay

The assay was implemented using the published Alamar Blue assay according to the published protocols [16,17]. Concisely, cancer cells, including P388, K562, HT-29, A549, and MOLT-4, were purchased from the American Type Culture Collection and individually seeded into a 96-well microtiter plate and incubated following the previously published procedure. The tested compounds were dissolved in DMSO and added to each well of cancer cells. After three days of culture, attached cells were treated with Alamar Blue for 4 h and subsequently measured at 595 nm using a microplate reader. 

### 3.6. Anti-Inflammatory Assay

Freshly isolated human neutrophils from blood using dextran sedimentation were incubated according to the published procedure [18,19]. The incubated neutrophils (6 × 10^5^ cells mL^−1^) were treated with compounds **1**–**3** in DMSO for 5 min. After the neutrophils were activated with fMLF (100 nM)/CB for 10 min, the amounts of superoxide generation and elastase release were measured at 550 nm and 405 nm, respectively, using a UV spectrometer apparatus.

## 4. Conclusions

Two cembranolides, namely tortuolides A and B (**1** and **2**), and a related cembranoid, namely *epi*-sarcophytonolide Q (**3**), were characterized from the persistent study of the soft coral *Sarcophyton tortuosum*. Compounds **1** and **2** are structurally related to emblide [12,20], featuring a C-8–C-20 α,β-unsaturated ε-lactone ring, and represented the first emblide-related cembranolide with a 1,2-epoxy functionality. The flexible nature of macrocyclic compounds, e.g. cembranoids, make the unambiguous assignment of chemical structures particularly challenging. In the present study, we showed the successful application of DFT calculations combined with statistical analysis methods, e.g. DP4+ and *J*-DP4, as well as the conventional NOESY approach. 

## Figures and Tables

**Table 1:** ^1^H and ^13^C NMR spectroscopic data of **1**–**3** in CDCl_3_.

	1 ^a^		2 ^a^		3 ^b^	
Position	*δ*_H_ (*J* in Hz)	*δ*_C_ (Type)	*δ*_H_ (*J* in Hz)	*δ*_C_ (Type)	*δ*_H_ (*J* in Hz)	*δ*_C_ (Type)
1		68.4 (qC)		68.4 (qC)		154.5 (qC)
2	3.28 d (10.0)	59.2 (CH)	3.81 d (8.5)	57.5 (CH)	6.92 d (11.2)	120.0 (CH)
3	6.69 d (10.0)	136.9 (CH)	6.48 d (8.5)	143.0 (CH)	6.64 d (11.2)	138.5 (CH)
4		136.3 (qC)		135.5 (qC)		126.5 (qC)
5	2.59 m	23.6 (CH_2_)	2.64 m	25.6 (CH_2_)	2.29 m	29.4 (CH_2_)
			2.71 m		2.62 m	
6	1.74 m	28.8 (CH_2_)	1.71 m	31.8 (CH_2_)	1.53 m	30.3 (CH_2_)
	2.37 m		2.42 m		2.02 t (12.4)	
7	4.93 dd (8.5, 2.0)	69.8 (CH)	4.80 br s	73.9 (CH)	3.70 d (10.4)	70.3 (CH)
8		83.5 (qC)		81.8 (qC)		74.6 (qC)
9	2.11 m	35.6 (CH_2_)	1.99 m	34.9 (CH_2_)	1.58 m	37.1 (CH_2_)
			2.11 m		2.16 m	
10	2.28 m	26.8 (CH_2_)	2.40 m	27.1 (CH_2_)	2.16 m	25.0 (CH_2_)
	2.43 m				2.74 m	
11	6.33 br s	140.4 (CH)	6.44 br s	139.2 (CH)	6.08 dd (8.0, 7.6)	144.5 (CH)
12		132.2 (qC)		131.3 (qC)		132.4 (qC)
13	2.34 m	29.4 (CH_2_)	2.20 m	34.9 (CH_2_)	2.38 m	33.14 (CH_2_)
	2.64 m		3.02 br d (14.0)		2.75 m	
14	1.82 m	28.2 (CH_2_)	1.90 td (14.0, 6.0)	26.4 (CH_2_)	2.30 m	30.1 (CH_2_)
	2.16 m		2.05 m		2.57 m	
15	1.84 m	32.2 (CH)	2.22 m	27.6 (CH)	2.46 m	33.07 (CH)
16	1.04 d (7.0)	19.4 (CH_3_)	0.89 d (6.5)	16.1 (CH_3_)	1.05 d (7.2)	22.25 (CH_3_)
17	1.12 d (6.5)	17.8 (CH_3_)	1.07 d (7.0)	17.8 (CH_3_)	1.10 d (7.2)	20.9 (CH_3_)
18		167.0 (qC)		166.8 (qC)		168.0 (qC)
19	1.52 s	24.5 (CH_3_)	1.39 s	24.2 (CH_3_)	1.12 s	22.34 (CH_3_)
20		167.6 (qC)		165.0 (qC)		168.5 (qC)
OAc	1.99 s	21.1 (CH_3_)	2.09 s	21.2 (CH_3_)		
		169.4 (qC)		170.3 (qC)		
18-OMe	3.73 s	52.0 (CH_3_)	3.78 s	52.0 (CH_3_)	3.75 s	51.2 (CH_3_)
20-OMe					3.62 s	51.6 (CH_3_)

^a^ Spectra were recorded at 500 (^1^H NMR) and 125 MHz (^13^C NMR). ^b^ Spectra were recorded at 400 (^1^H NMR) and 100 MHz (^13^C NMR).

**Table 2:** *J*-DP4 (PCM/B3LYP/6-31+G(d,p)) probabilities for compounds **1** and **2**.

			*J*-DP4 (%)		
	1R*,2S*,7S*,8R*-1	1R*,2S*,7R*,8R*-1	1R*,2R*,7R*,8R*-2	1R*,2R*,7S*,8R*-2	1R*,2R*,7R*,8S*-2
H	0.06	99.94	3.38	96.62	0
C	100.00	0	0.02	97.52	2.46
H + C	99.16	0.84	0	100.00	0
*J*	61.32	38.68	1.63	11.93	86.43
all data	99.47	0.53	0	100.00	0

**Table 3:** DP4+ (PCM/mPW1PW91/6-31+G(d,p)) probabilities for compound **3**.

		DP4 + (%)	
	H	C	All Data
7S*,8R*-3	99.75	100.00	100.00
7R*,8R*-3	0.25	0	0

## Data Availability

Data of the present study are available in the article and Supplementary Materials.

## Supplementary Materials

The following supporting information can be downloaded at: https://www.mdpi.com/article/10.3390/md20050297/s1, Figure S1: LR- and HR-ESIMS spectra of **1**, Figure S2: ^1^H NMR spectrum of **1** in CDCl_3_, Figure S3: ^13^C NMR spectrum of **1** in CDCl_3_, Figure S4: DEPT and ^13^C NMR spectra of **1** in CDCl_3_, Figure S5: HSQC spectrum of **1** in CDCl_3_, Figure S6: ^1^H–^1^H COSY spectrum of **1** in CDCl_3_, Figure S7: HMBC spectrum of **1** in CDCl_3_, Figure S8: NOESY spectrum of **1** in CDCl_3_, Figure S9: LR- and HR-ESIMS spectra of **2**, Figure S10: ^1^H NMR spectrum of **2** in CDCl_3_, Figure S11: ^13^C NMR spectrum of **2** in CDCl_3_, Figure S12: DEPT and ^13^C NMR spectra of **2** in CDCl_3_, Figure S13: HSQC spectrum of **2** in CDCl_3_, Figure S14: ^1^H–^1^H COSY spectrum of **2** in CDCl_3_, Figure S15: HMBC spectrum of **2** in CDCl_3_, Figure S16: NOESY spectrum of **2** in CDCl_3_, Figure S17: LR- and HR-ESIMS spectra of **3**, Figure S18: ^1^H NMR spectrum of **3** in CDCl_3_, Figure S19: ^13^C NMR spectrum of **3** in CDCl_3_, Figure S20: DEPT and ^13^C NMR spectra of **3** in CDCl_3_, Figure S21: HSQC spectrum of **3** in CDCl_3_, Figure S22: ^1^H–^1^H COSY spectrum of **3** in CDCl_3_, Figure S23: HMBC spectrum of **3** in CDCl_3_, Figure S24: NOESY spectrum of **3** in CDCl_3_, Table S1. Low-energy conformers of 1*R*,2*S*,7*S*,8*R*-**1** for ECD calculations, Table S2: Low-energy conformers of **2** for ECD calculations, Table S3: Conformers of 1*R**,2*S**,7*S**,8*S**-**1** for *J*-DP4 calculation and the dihedral angles of selected protons at MMFF94 level, Table S4: Conformers of 1*R**,2*S**,7*R**,8*S**-**1** for *J*-DP4 calculation and the dihedral angles of selected protons at MMFF94 level, Table S5: Conformers of 1*R**,2*S**,7*S**,8*R**-**1** for *J*-DP4 calculation and the dihedral angles of selected protons at MMFF94 level, Table S6: Conformers of 1*R**,2*S**,7*R**,8*R**-**1** for *J*-DP4 calculation and the dihedral angles of selected protons at MMFF94 level, Table S7: Conformers of 1*R**,2*R**,7*R**,8*R**-**2** for *J*-DP4 calculation and the dihedral angles of selected protons at MMFF94 level, Table S8: Conformers of 1*R**,2*R**,7*S**,8*R**-**2** for *J*-DP4 calculation and the dihedral angles of selected protons at MMFF94 level, Table S9: Conformers of 1*R**,2*R**,7*R**,8*S**-**2** for *J*-DP4 calculation and the dihedral angles of selected protons at MMFF94 level, Table S10: Conformers of 1*R**,2*R**,7S***,8S***-**2** for *J*-DP4 calculation and the dihedral angles of selected protons at MMFF94 level. Tables S3–S10 [21].
