# Supplementary material for: Computationally Assisted Structural Elucidation of Cembranoids from the Soft Coral Sarcophyton tortuosum
Source: Mar Drugs. 2022 Apr 27;20(5):297. doi: 10.3390/md20050297 (PMC9147035; doi:10.3390/md20050297)
Supplement: Supplementary file 1 [file marinedrugs-20-00297-s001.zip › marinedrugs-1664509-supplementary.pdf]

Computationally Assisted Structural Elucidation of Cembranoids from the Soft Coral *Sarcophyton tortuosum*

Chih-Hua Chao [1,2,†], Kuan-Hua Lin [3,†], Chiung-Yao Huang [3], Tsong-Long Hwang [4,5,6,7,8], Chang-Feng Dai [9], Hui-Chi Huang [10] and Jyh-Horng Sheu [3,11,12,*]

[1] School of Pharmacy, China Medical University, Taichung 406040, Taiwan; chchao@mail.cmu.edu.tw
[2] Chinese Medicine Research and Development Center, China Medical University Hospital, Taichung 404332, Taiwan
[3] Department of Marine Biotechnology and Resources, National Sun Yat-sen University, Kaohsiung 80424, Taiwan; m005020002@student.nsysu.edu.tw (K.-H.L.); betty8575@yahoo.com.tw (C.-Y.H.)
[4] Graduate Institute of Natural Products, College of Medicine, Chang Gung University, Taoyuan 33303, Taiwan, Taiwan; htl@mail.cgu.edu.tw
[5] Research Center for Chinese Herbal Medicine, College of Human Ecology, Chang Gung University of Science and Technology, Taoyuan 33303, Taiwan
[6] Research Center for Food and Cosmetic Safety, College of Human Ecology, Chang Gung University of Science and Technology, Taoyuan 33303, Taiwan
[7] Graduate Institute of Health Industry Technology, College of Human Ecology, Chang Gung University of Science and Technology, Taoyuan 33303, Taiwan
[8] Department of Anesthesiology, Chang Gung Memorial Hospital, Taoyuan 33303, Taiwan
[9] Institute of Oceanography, National Taiwan University, Taipei 10617, Taiwan; corallab@ntu.edu.tw
[10] Department of Chinese Pharmaceutical Sciences and Chinese Medicine Resources, China Medical University, Taichung, 40402, Taiwan; hchuang@mail.cmu.edu.tw
[11] Department of Medical Research, China Medical University Hospital, China Medical University, Taichung 404332, Taiwan
[12] Graduate Institute of Natural Products, College of Pharmacy, Kaohsiung Medical University, Kaohsiung 80708, Taiwan

*Correspondence: sheu@mail.nsysu.edu.tw; Tel: +886-7-5252000 (ext. 5030); Fax: +886-7-5255020
† These authors contributed equally to this work

Figure S1. LR- and HR-ESIMS spectra of **1**.
Figure S2. ^{1}H NMR spectrum of **1** in $CDCl_3$.
Figure S3. ^{13}C NMR spectrum of **1** in $CDCl_3$.
Figure S4. DEPT and ^{13}C NMR spectra of **1** in $CDCl_3$.
Figure S5. HSQC spectrum of **1** in $CDCl_3$.
Figure S6. ^{1}H–^{1}H COSY spectrum of **1** in $CDCl_3$.
Figure S7. HMBC spectrum of **1** in $CDCl_3$.
Figure S8. NOESY spectrum of **1** in $CDCl_3$.
Figure S9. LR- and HR-ESIMS spectra of **2**.
Figure S10. ^{1}H NMR spectrum of **2** in $CDCl_3$.
Figure S11. ^{13}C NMR spectrum of **2** in $CDCl_3$.
Figure S12. DEPT and ^{13}C NMR spectra of **2** in $CDCl_3$.
Figure S13. HSQC spectrum of **2** in $CDCl_3$.
Figure S14. ^{1}H–^{1}H COSY spectrum of **2** in $CDCl_3$.
Figure S15. HMBC spectrum of **2** in $CDCl_3$.
Figure S16. NOESY spectrum of **2** in $CDCl_3$.
Figure S17. LR- and HR-ESIMS spectra of **3**.
Figure S18. ^{1}H NMR spectrum of **3** in $CDCl_3$.
Figure S19. ^{13}C NMR spectrum of **3** in $CDCl_3$.
Figure S20. DEPT and ^{13}C NMR spectra of **3** in $CDCl_3$.
Figure S21. HSQC spectrum of **3** in $CDCl_3$.
Figure S22. ^{1}H–^{1}H COSY spectrum of **3** in $CDCl_3$.
Figure S23. HMBC spectrum of **3** in $CDCl_3$.
Figure S24. NOESY spectrum of **3** in $CDCl_3$.
Table S1. Low-energy conformers of 1*R*,2*S*,7*S*,8*R*-**1** for ECD calculations.
Table S2. Low-energy conformers of **2** for ECD calculations.
Table S3. Conformers of 1*R**,2*S**,7*S**,8*S**-**1** for *J*-DP4 calculation and the dihedral angles of selected protons at MMFF94 level (the selected conformers were marked in yellow).
Table S4. Conformers of 1*R**,2*S**,7*R**,8*S**-**1** for *J*-DP4 calculation and the dihedral angles of selected protons at MMFF94 level (the selected conformers were marked in yellow).
Table S5. Conformers of 1*R**,2*S**,7*S**,8*R**-**1** for *J*-DP4 calculation and the dihedral angles of selected protons at MMFF94 level (the selected conformers were marked in yellow).
Table S6. Conformers of 1*R**,2*S**,7*R**,8*R**-**1** for *J*-DP4 calculation and the dihedral angles of selected protons at MMFF94 level (the selected conformers were marked in yellow).

Table S7. Conformers of 1*R**,2*R**,7*R**,8*R**-**2** for *J*-DP4 calculation and the dihedral angles of selected protons at MMFF94 level (the selected conformers were marked in yellow).

Table S8. Conformers of 1*R**,2*R**,7*S**,8*R**-**2** for *J*-DP4 calculation and the dihedral angles of selected protons at MMFF94 level (the selected conformers were marked in yellow).

Table S9. Conformers of 1*R**,2*R**,7*R**,8*S**-**2** for *J*-DP4 calculation and the dihedral angles of selected protons at MMFF94 level (the selected conformers were marked in yellow).

Table S10. Conformers of 1*R**,2*R**,7S*,8S*-**2** for *J*-DP4 calculation and the dihedral angles of selected protons at MMFF94 level (none of the conformers was selected).

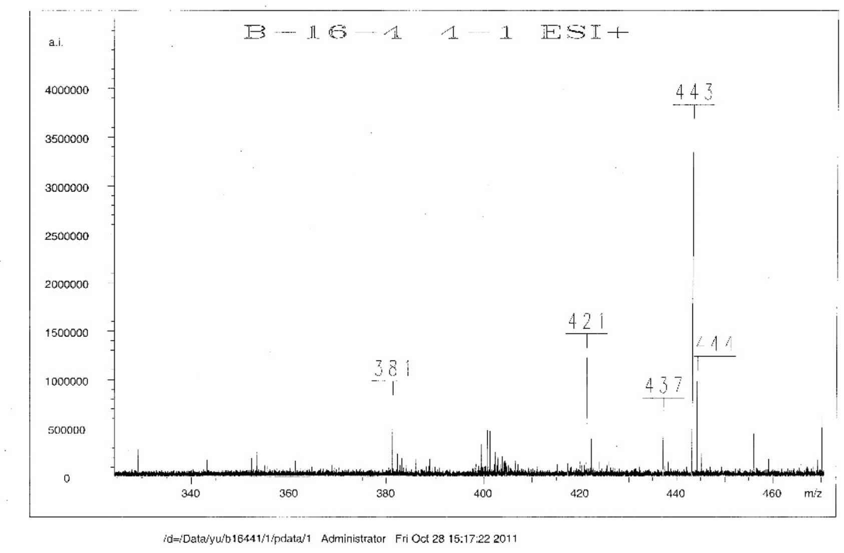

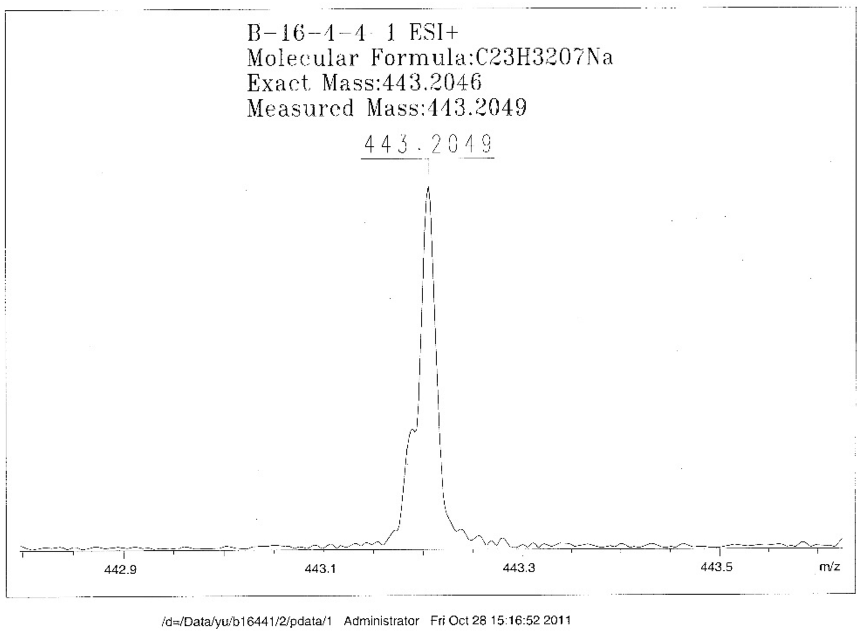

Figure S1. LR- and HR-ESIMS spectra of **1**.

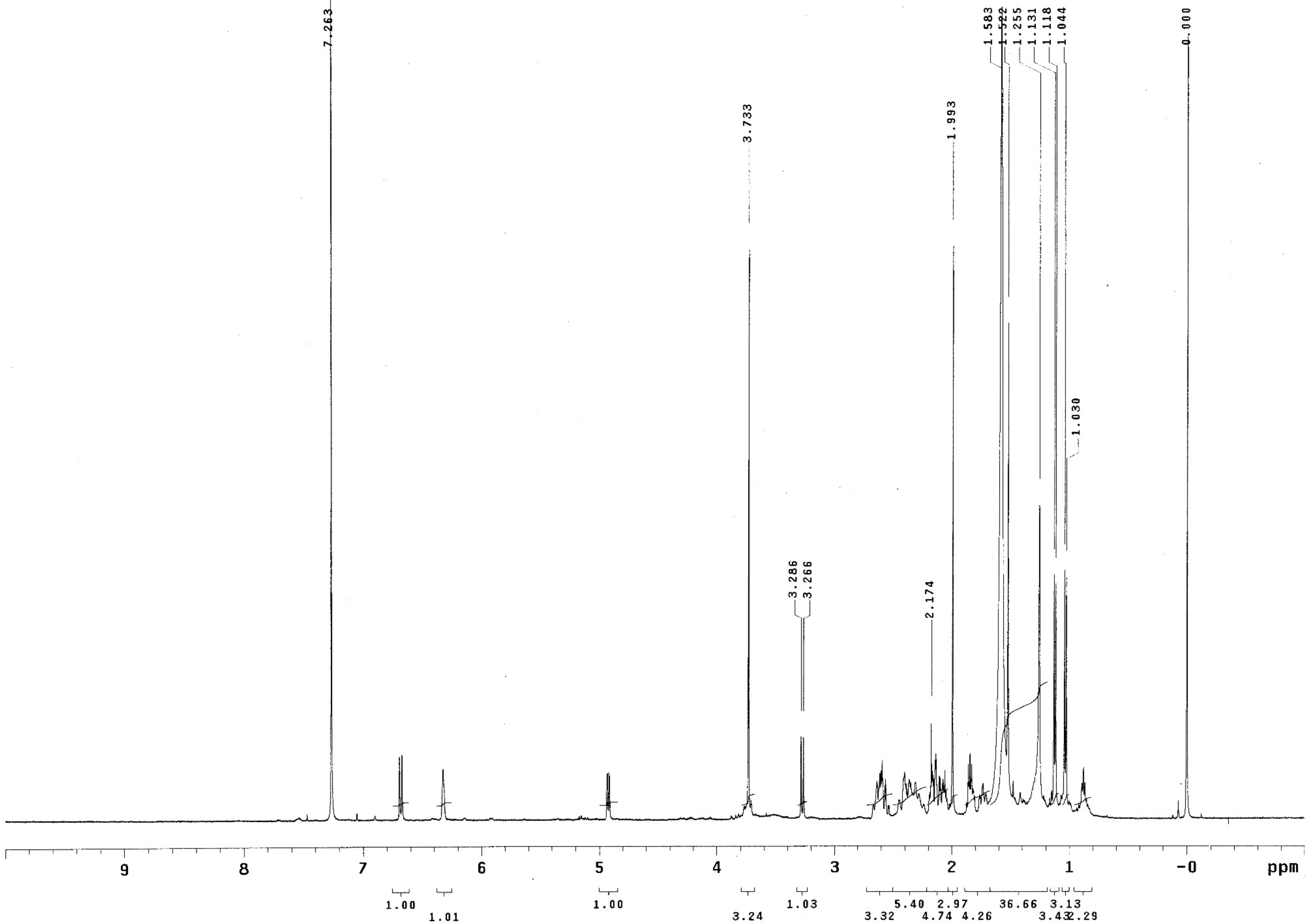

Figure S2. ^{1}H NMR spectrum of **1** in $CDCl_3$.

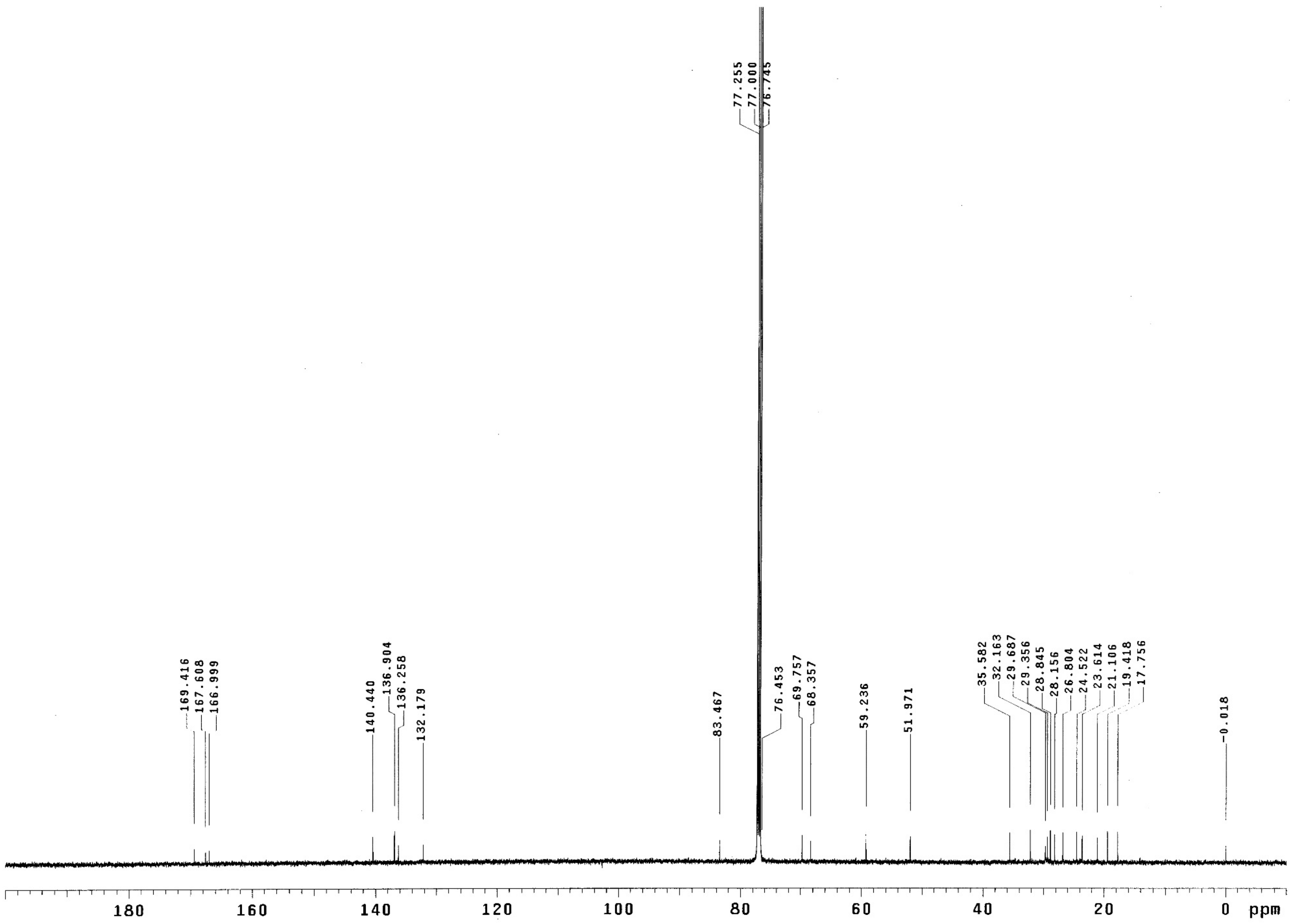

Figure S3. ^{13}C NMR spectrum of **1** in $CDCl_3$.

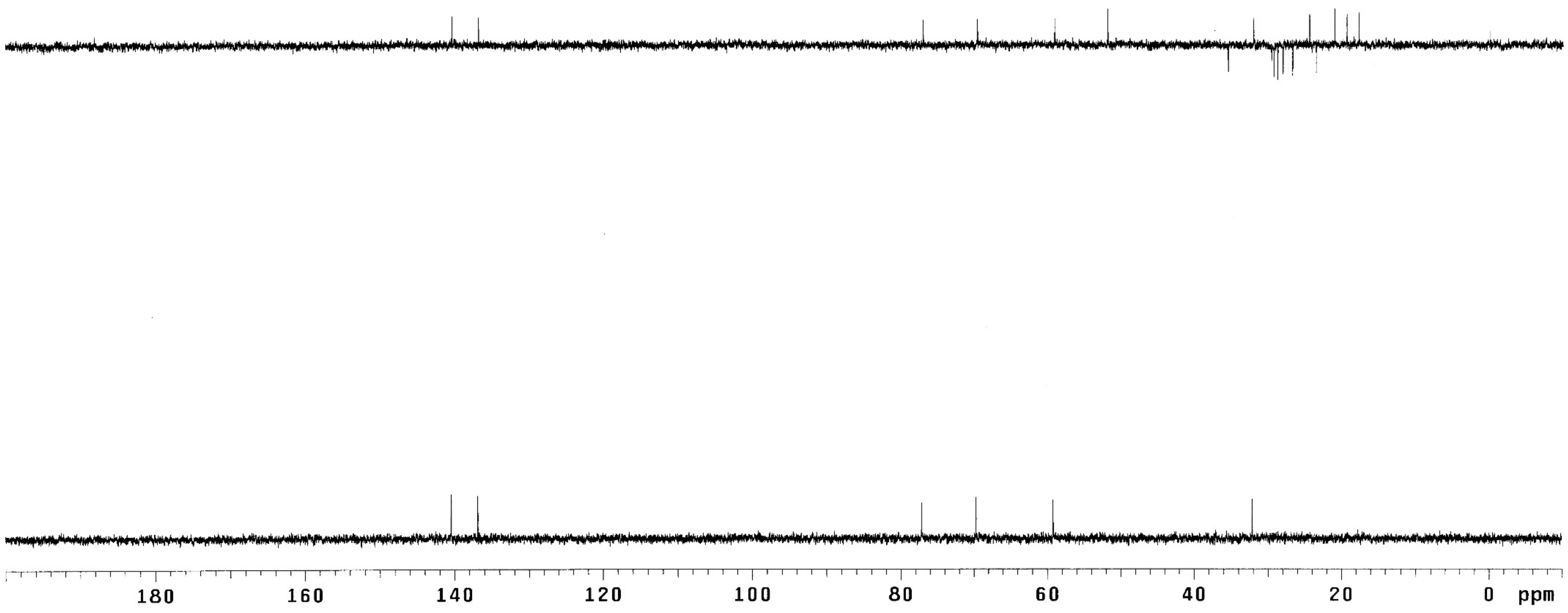

Figure S4. DEPT and ^{13}C NMR spectra of **1** in $CDCl_3$.

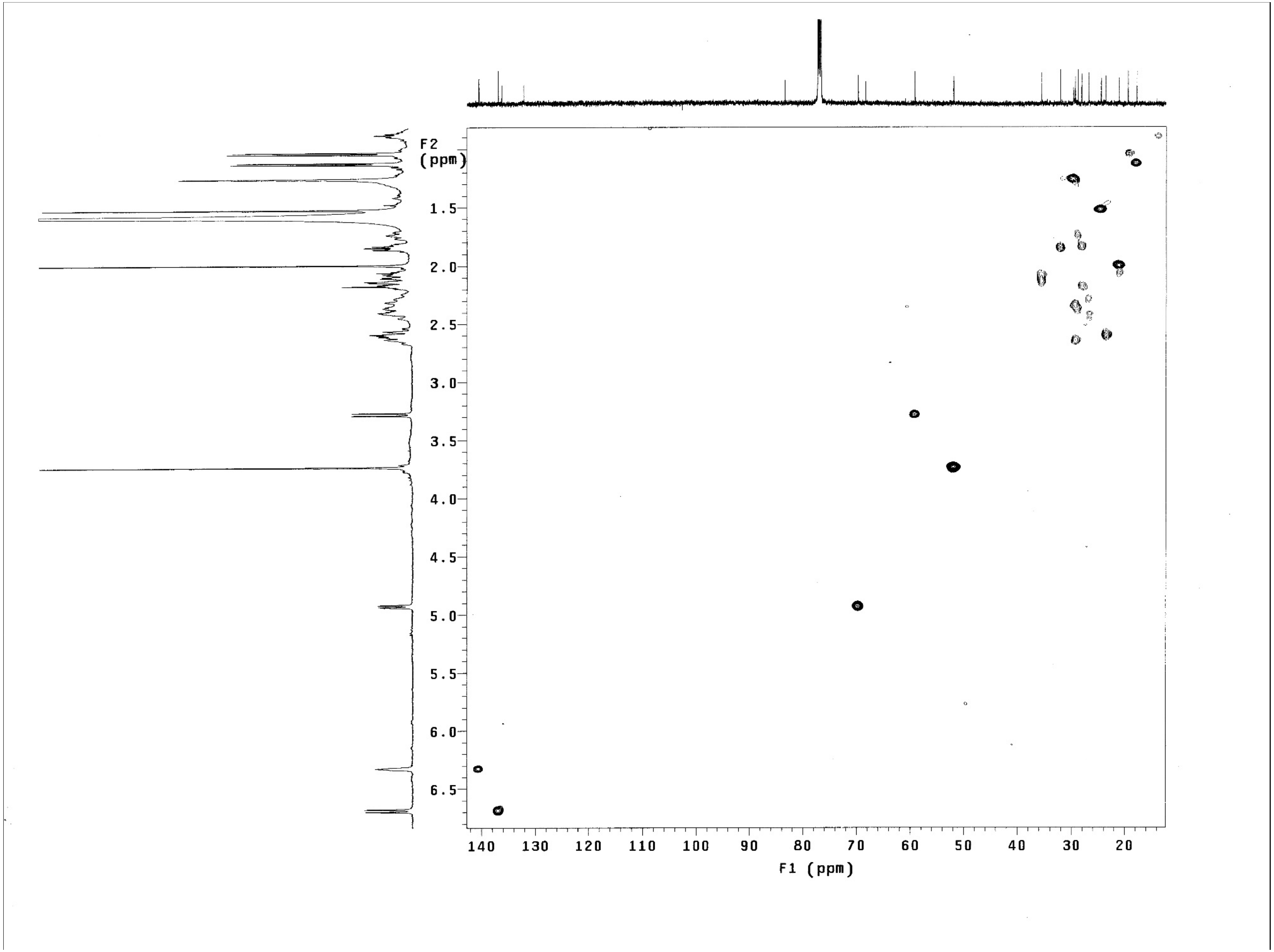

Figure S5. HSQC spectrum of **1** in $CDCl_3$.

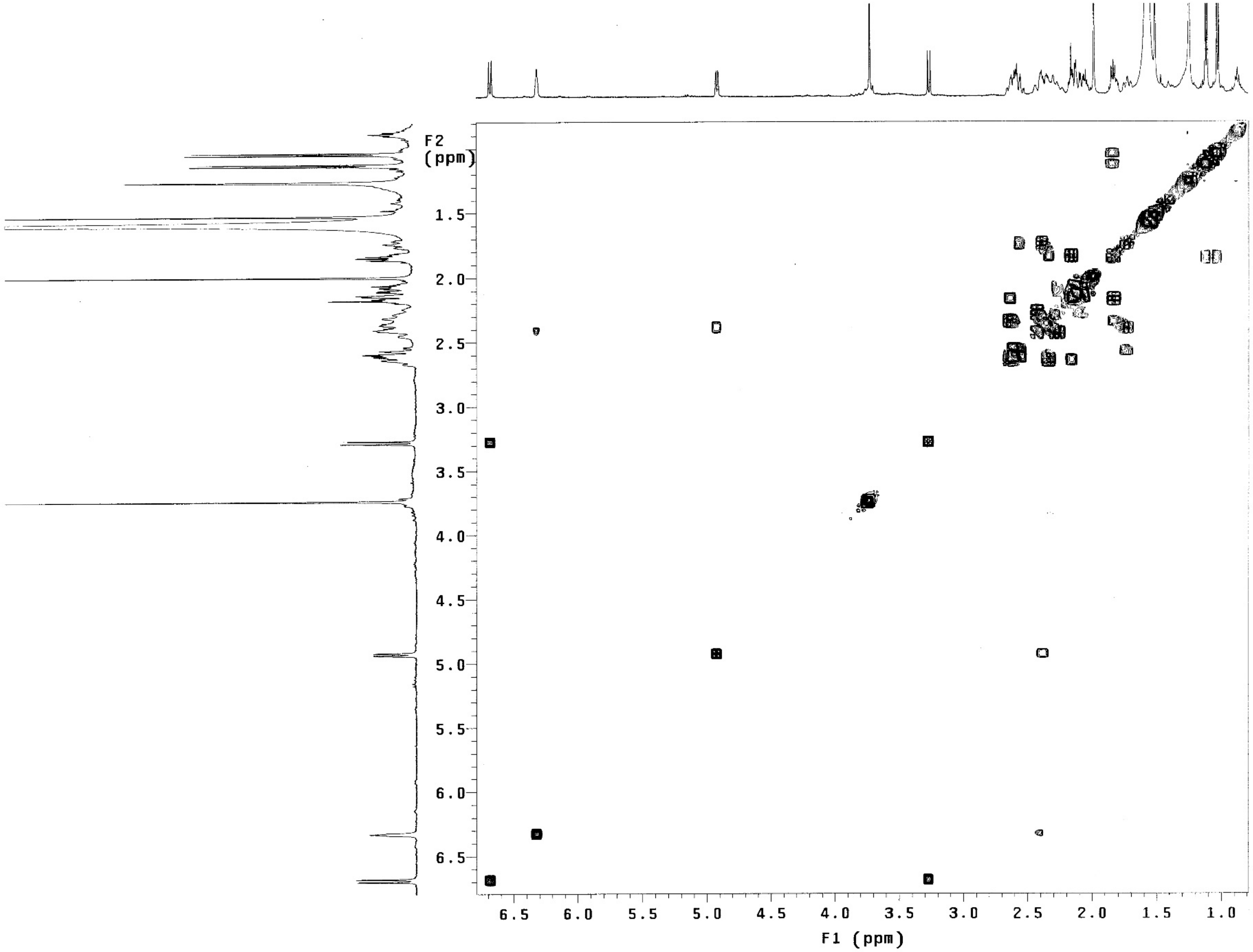

Figure S6. ^{1}H–^{1}H COSY spectrum of **1** in $CDCl_3$.

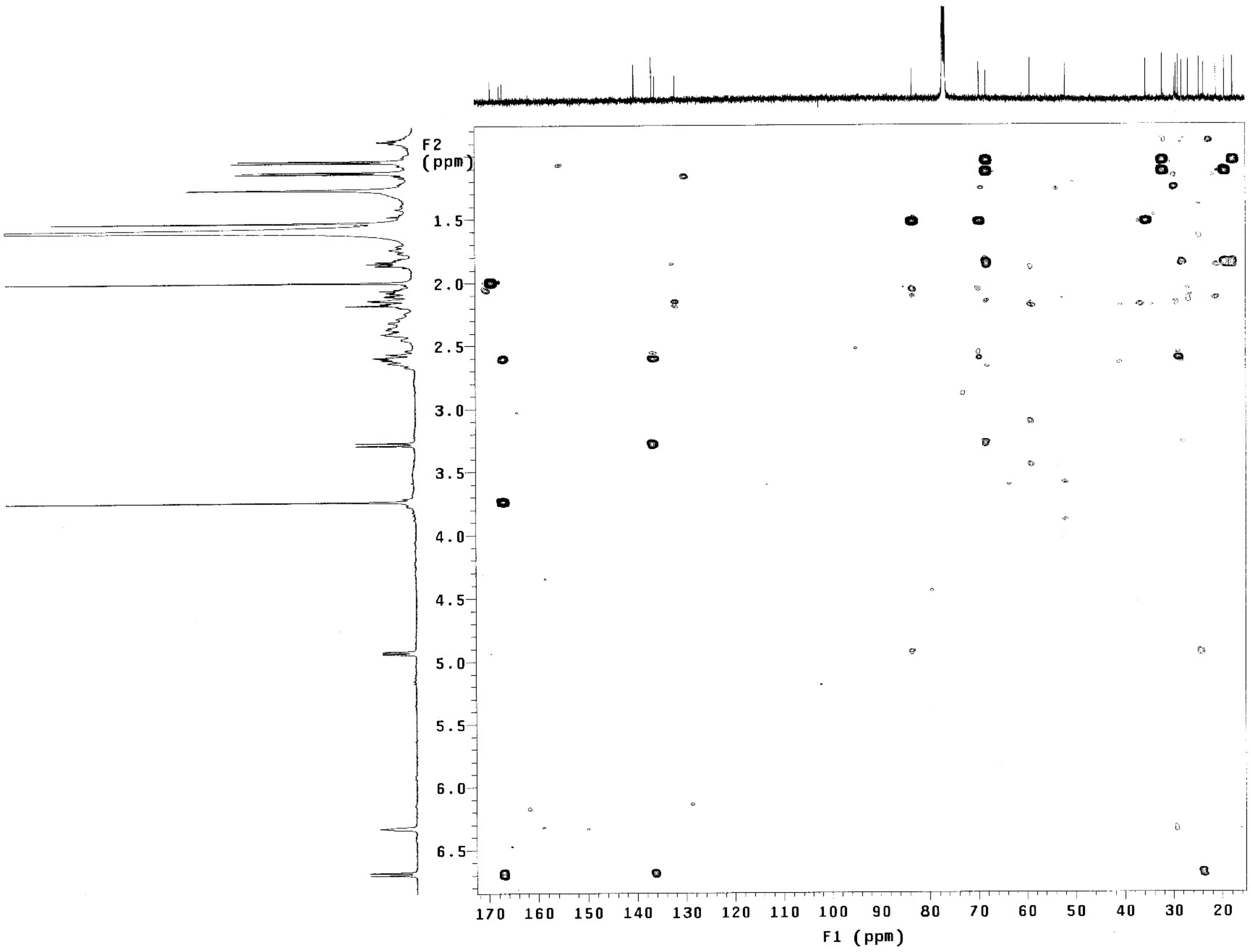

Figure S7. HMBC spectrum of **1** in $CDCl_3$.

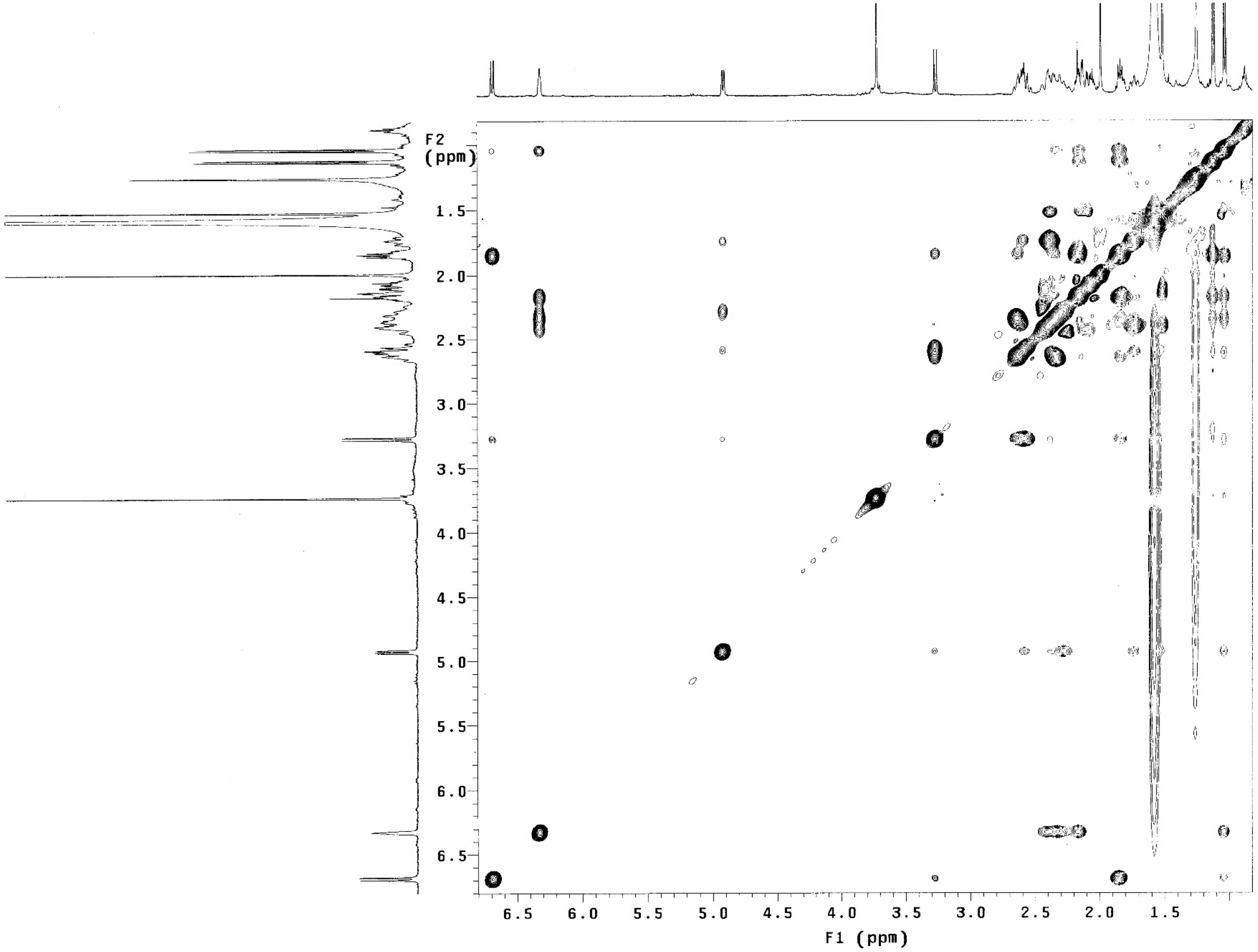

Figure S8. NOESY spectrum of **1** in $CDCl_3$.

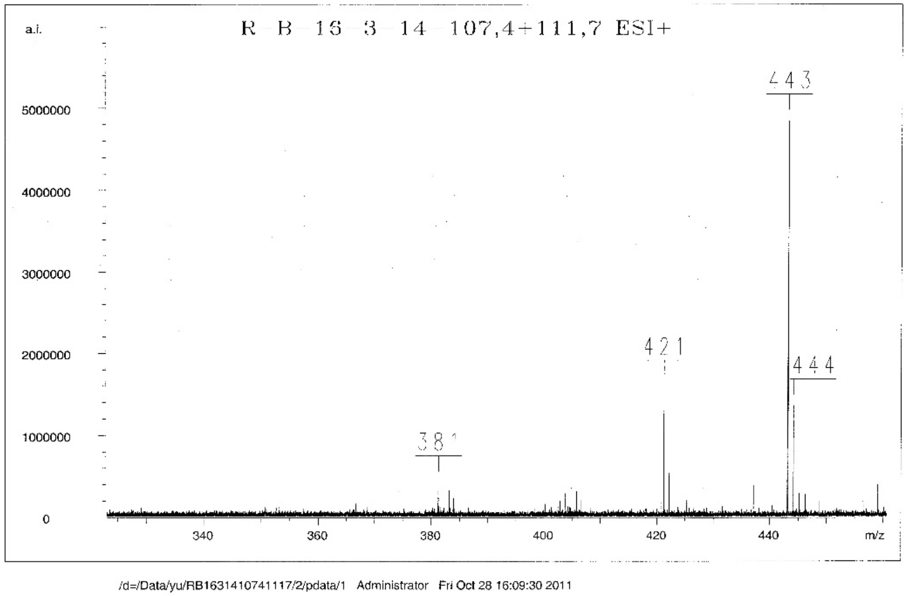

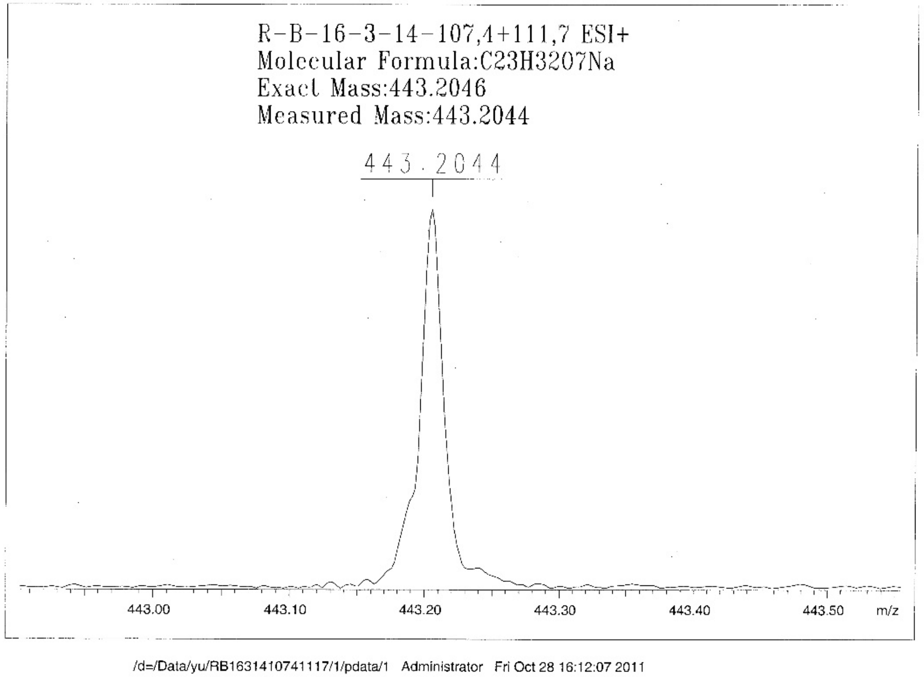

Figure S9. LR- and HR-ESIMS spectra of **2**.

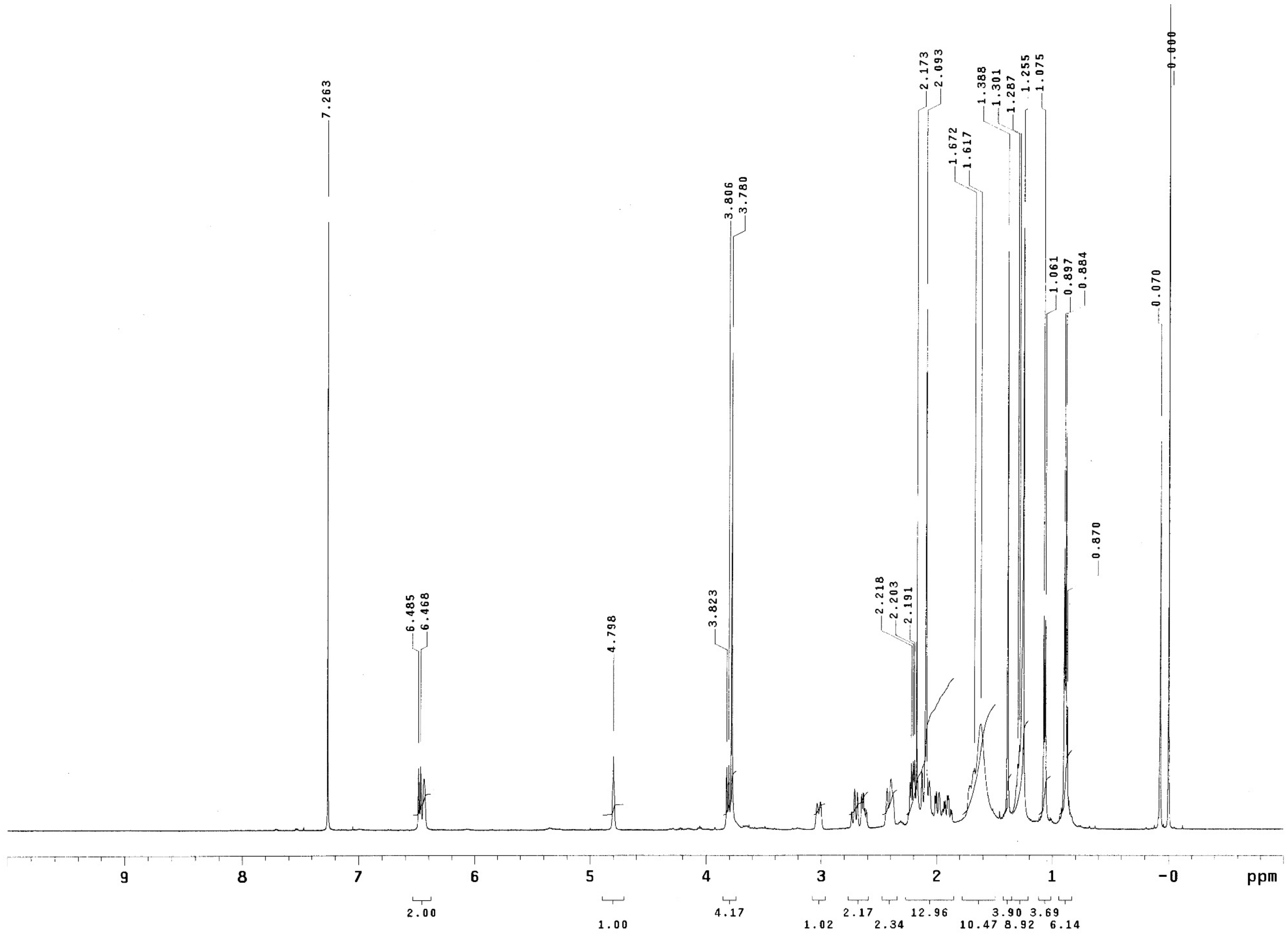

Figure S10. ^{1}H NMR spectrum of **2** in $CDCl_3$.

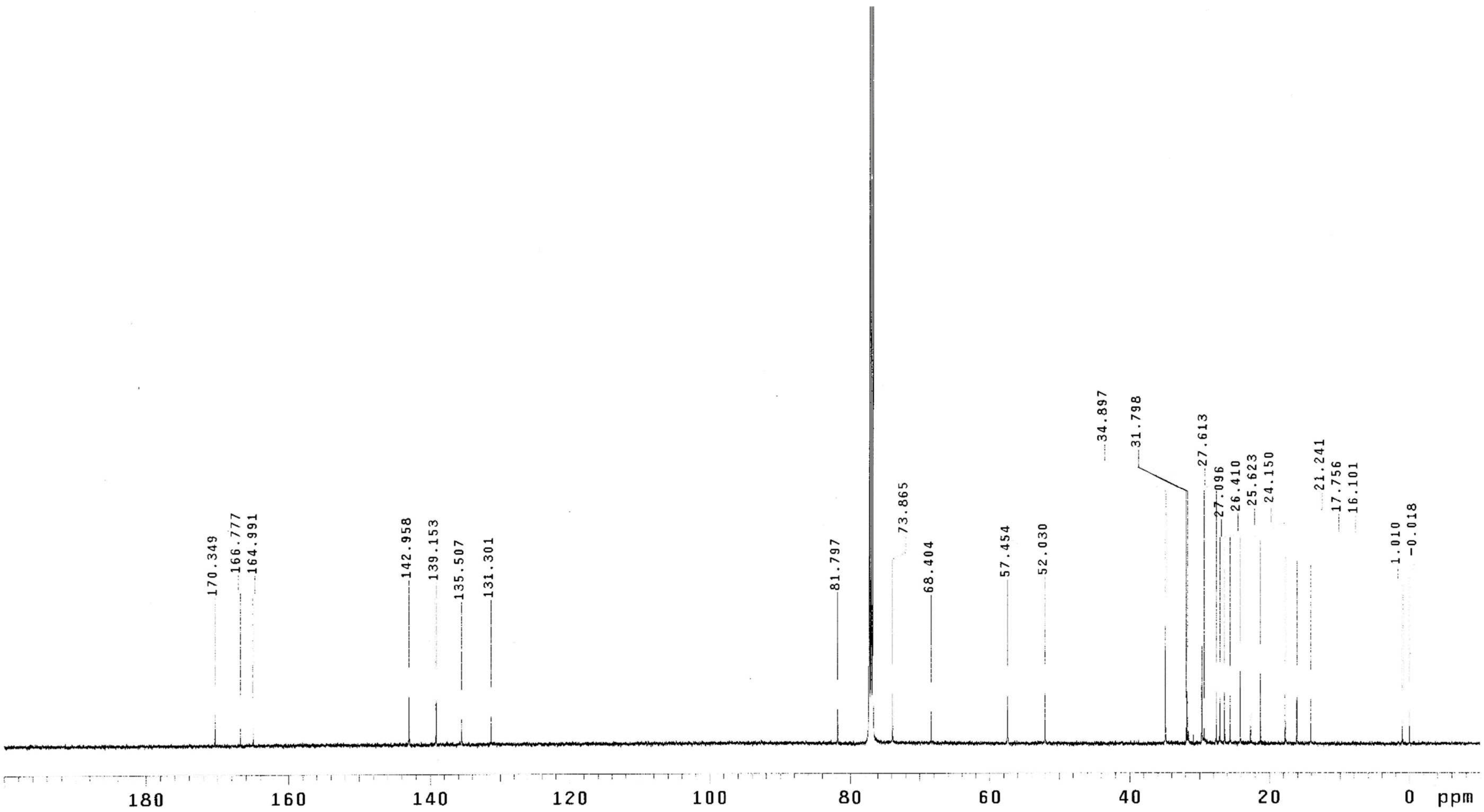

Figure S11. ^{13}C NMR spectrum of **2** in $CDCl_3$.

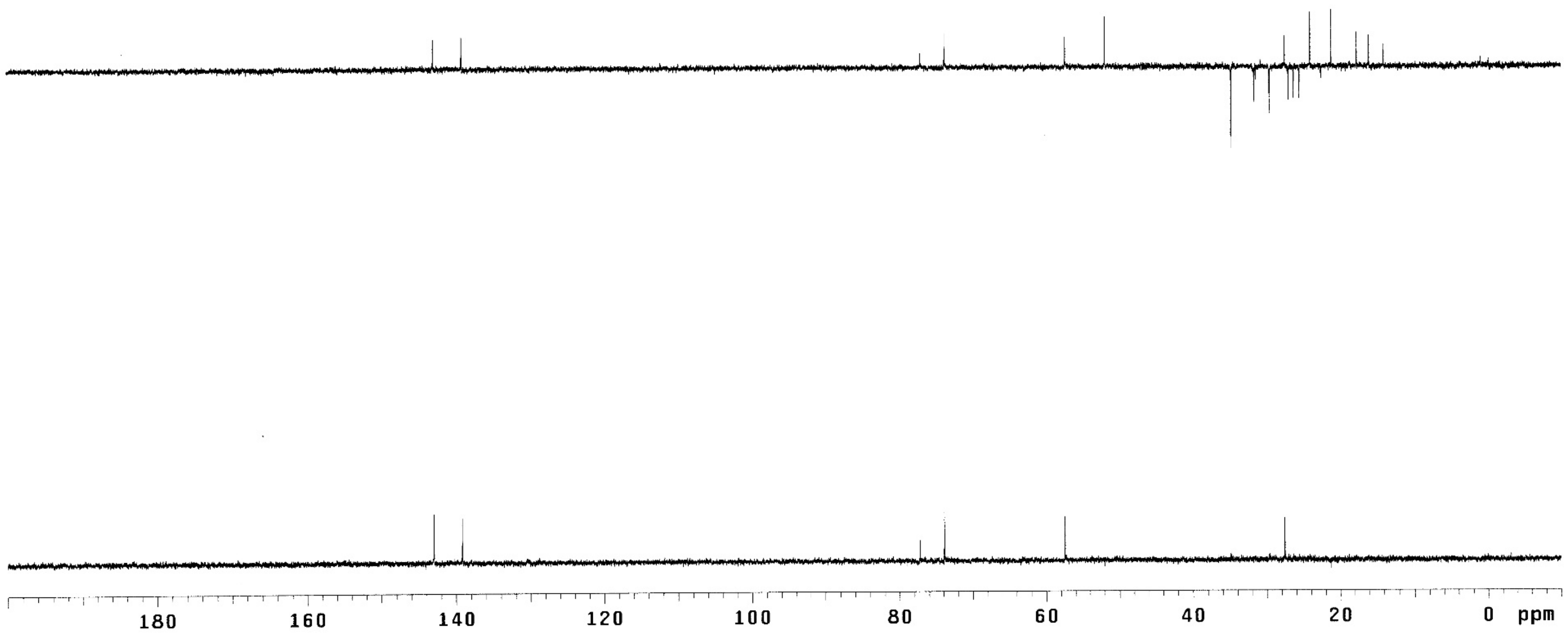

Figure S12. DEPT and ^{13}C NMR spectra of **2** in $CDCl_3$.

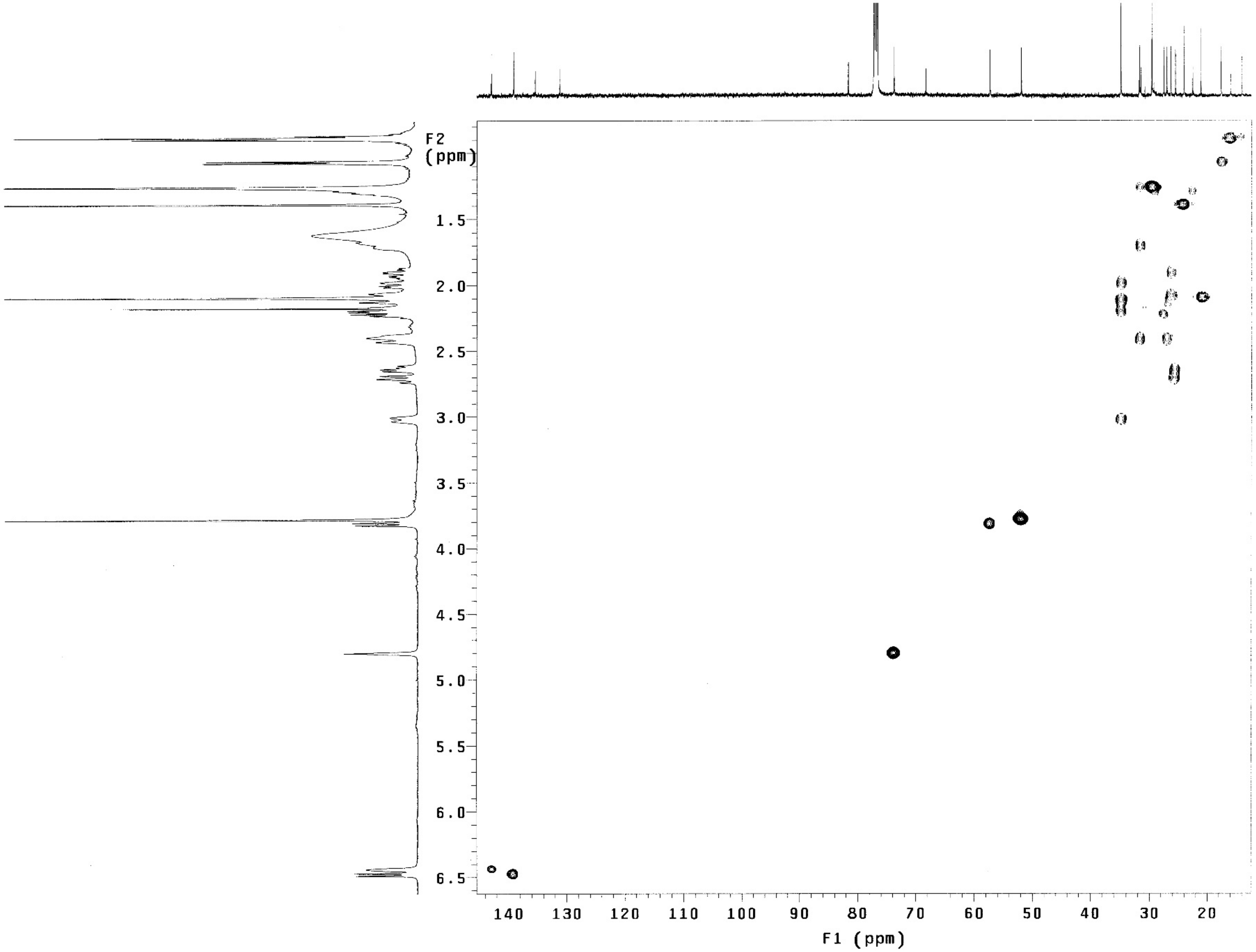

Figure S13. HSQC spectrum of **2** in $CDCl_3$.

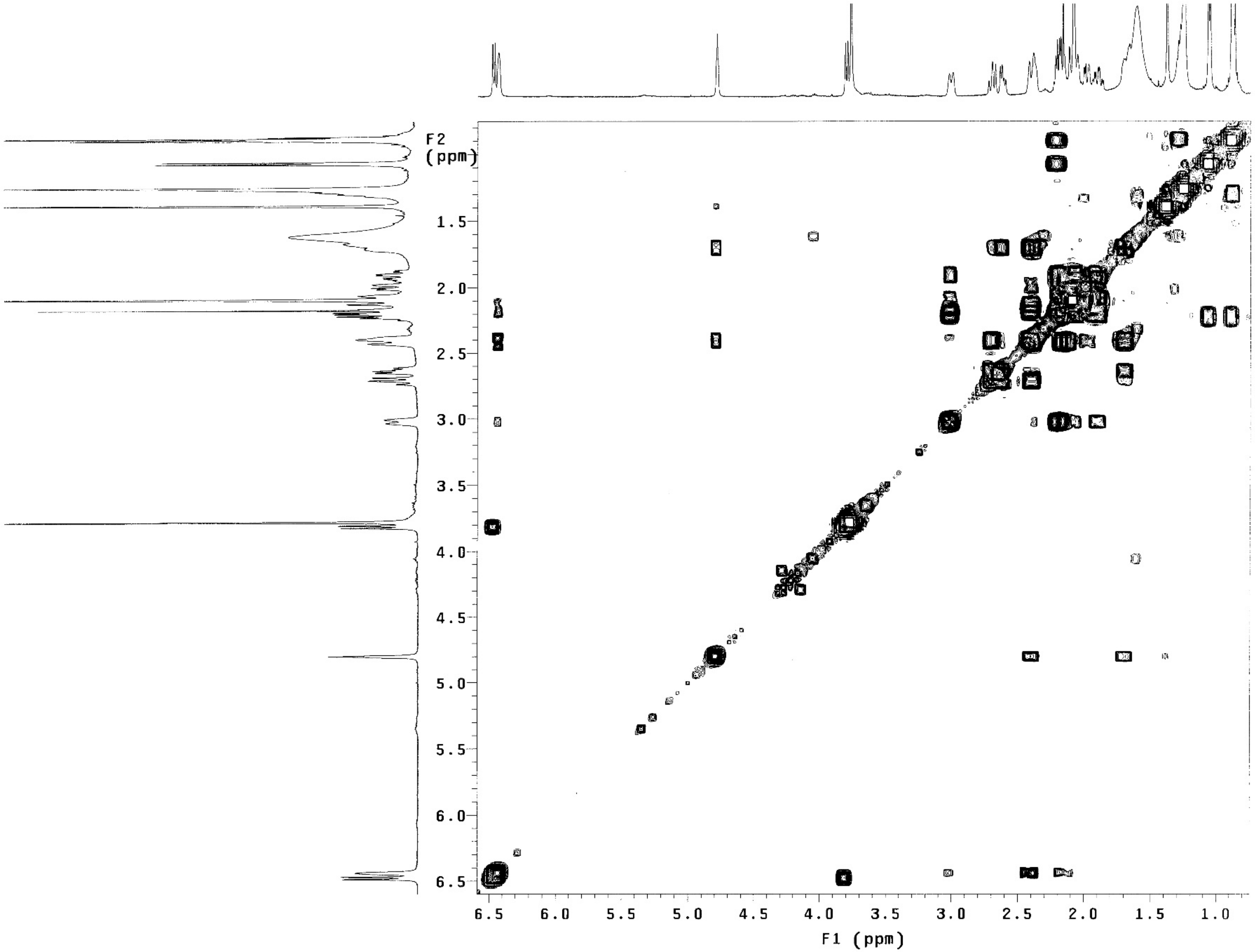

Figure S14. ^{1}H–^{1}H COSY spectrum of **2** in $CDCl_3$.

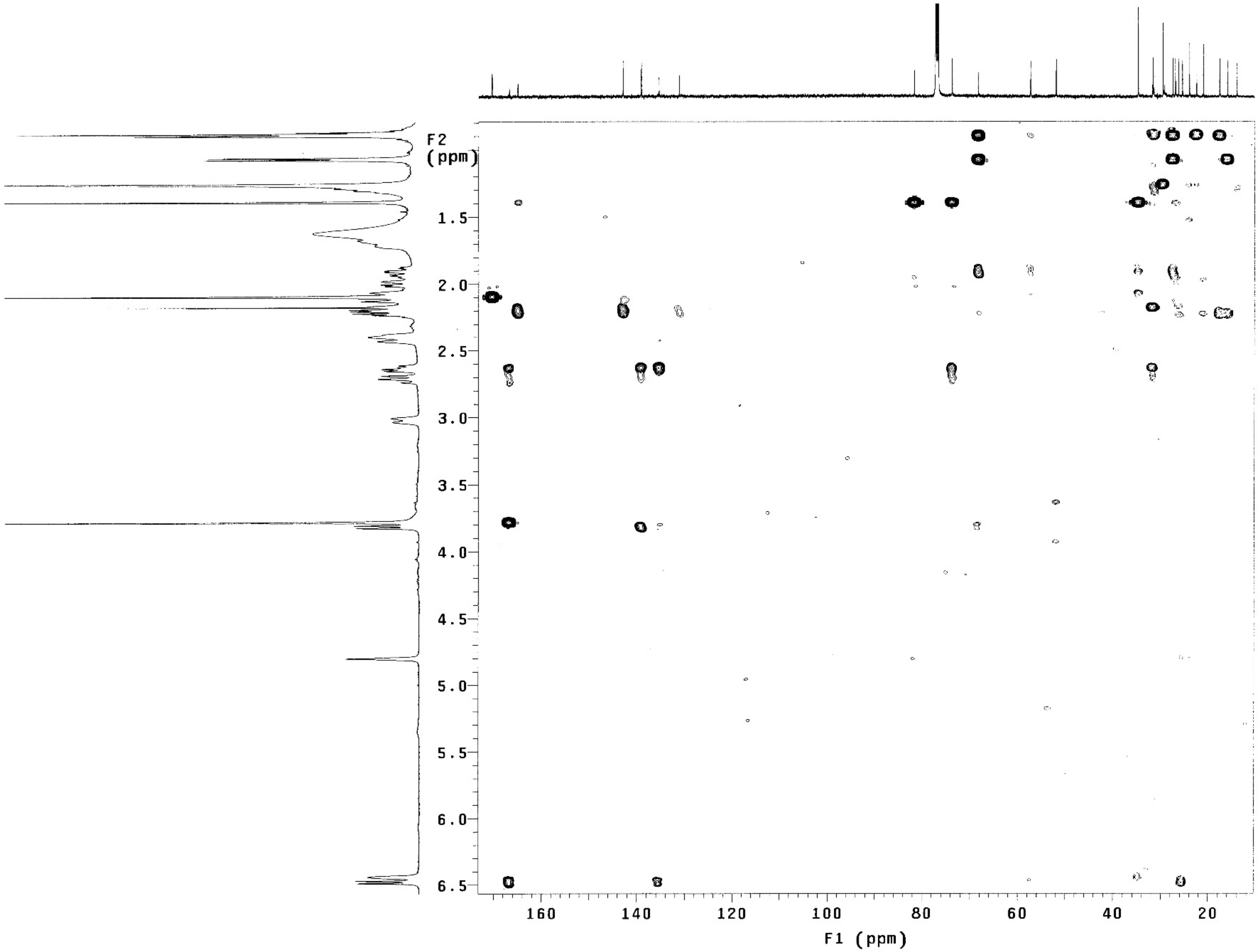

Figure S15. HMBC spectrum of **2** in $CDCl_3$.

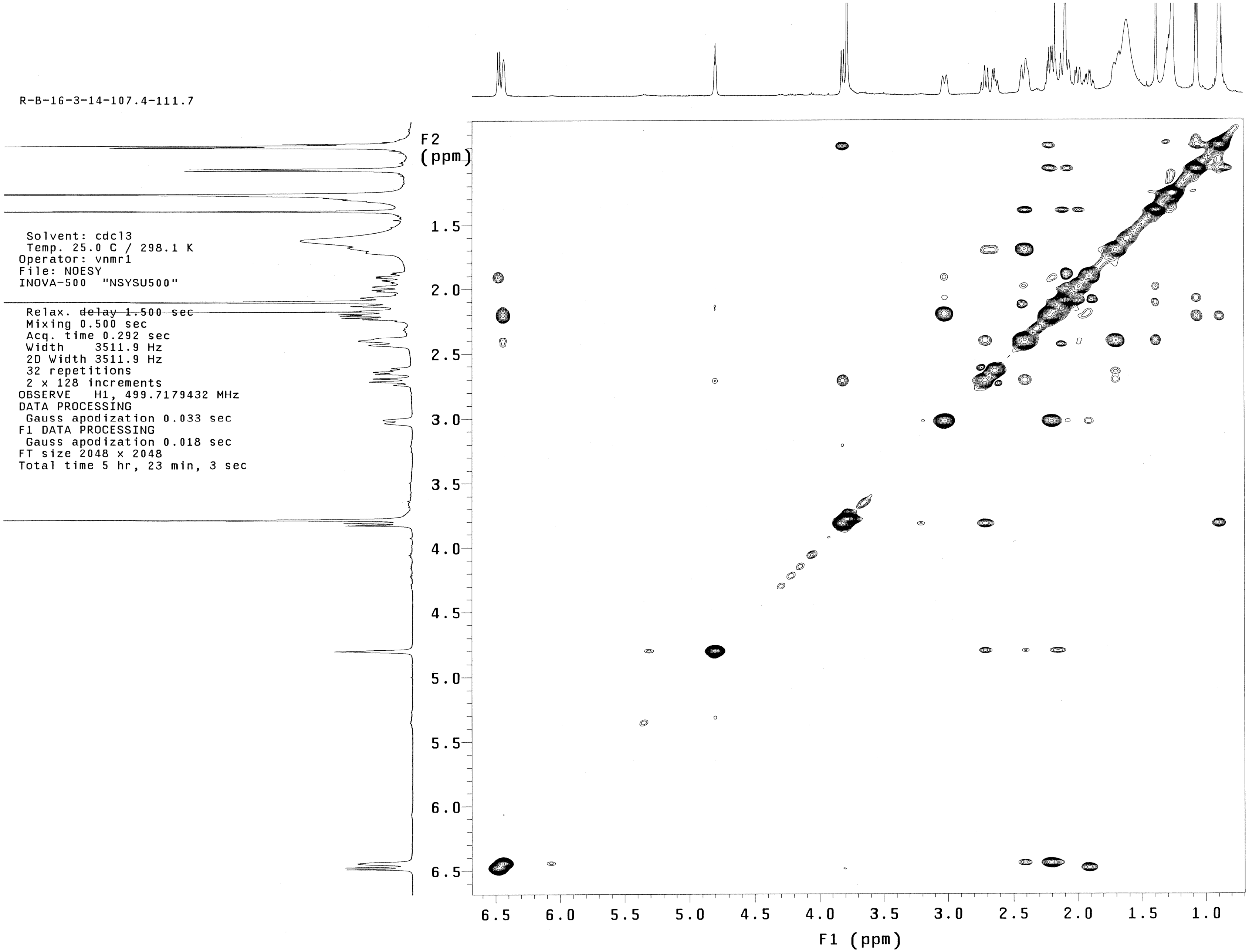

Figure S16. NOESY spectrum of **2** in $CDCl_3$.

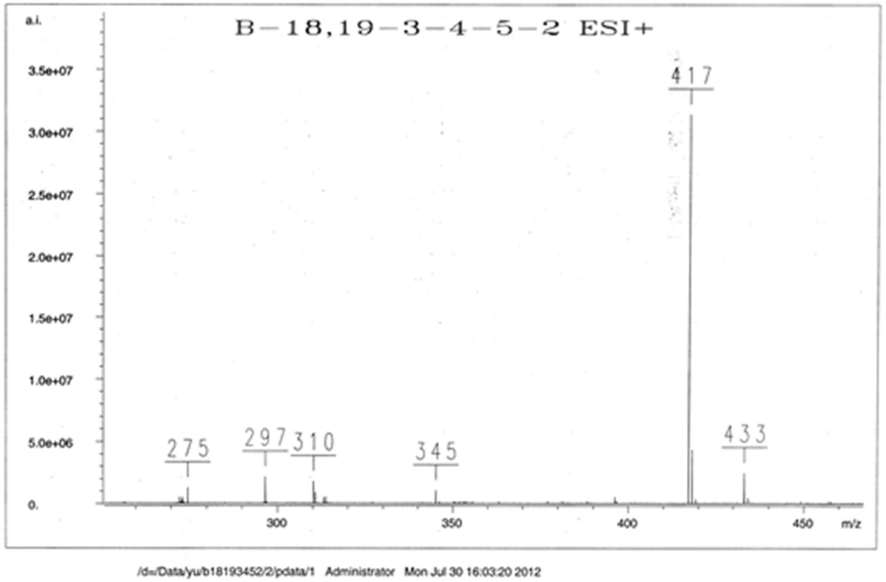

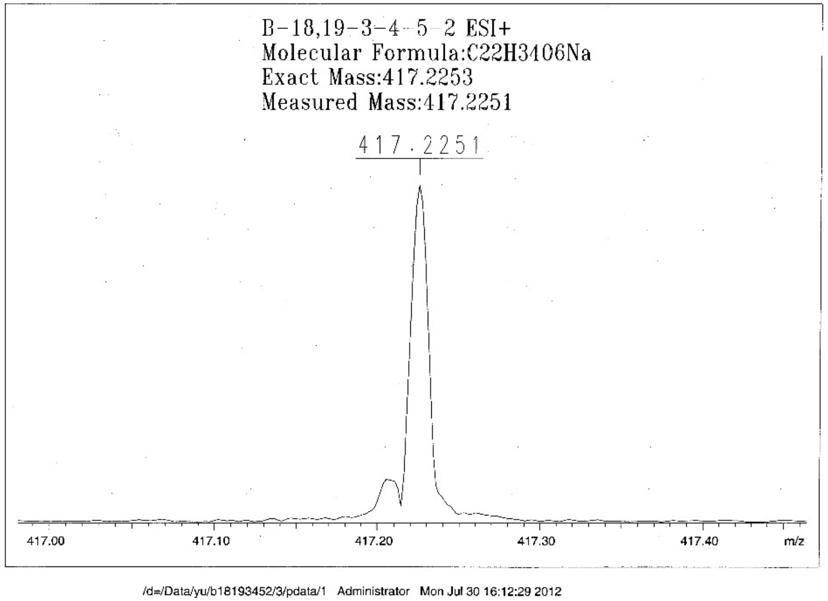

Figure S17. LR- and HR-ESIMS spectra of **3**.

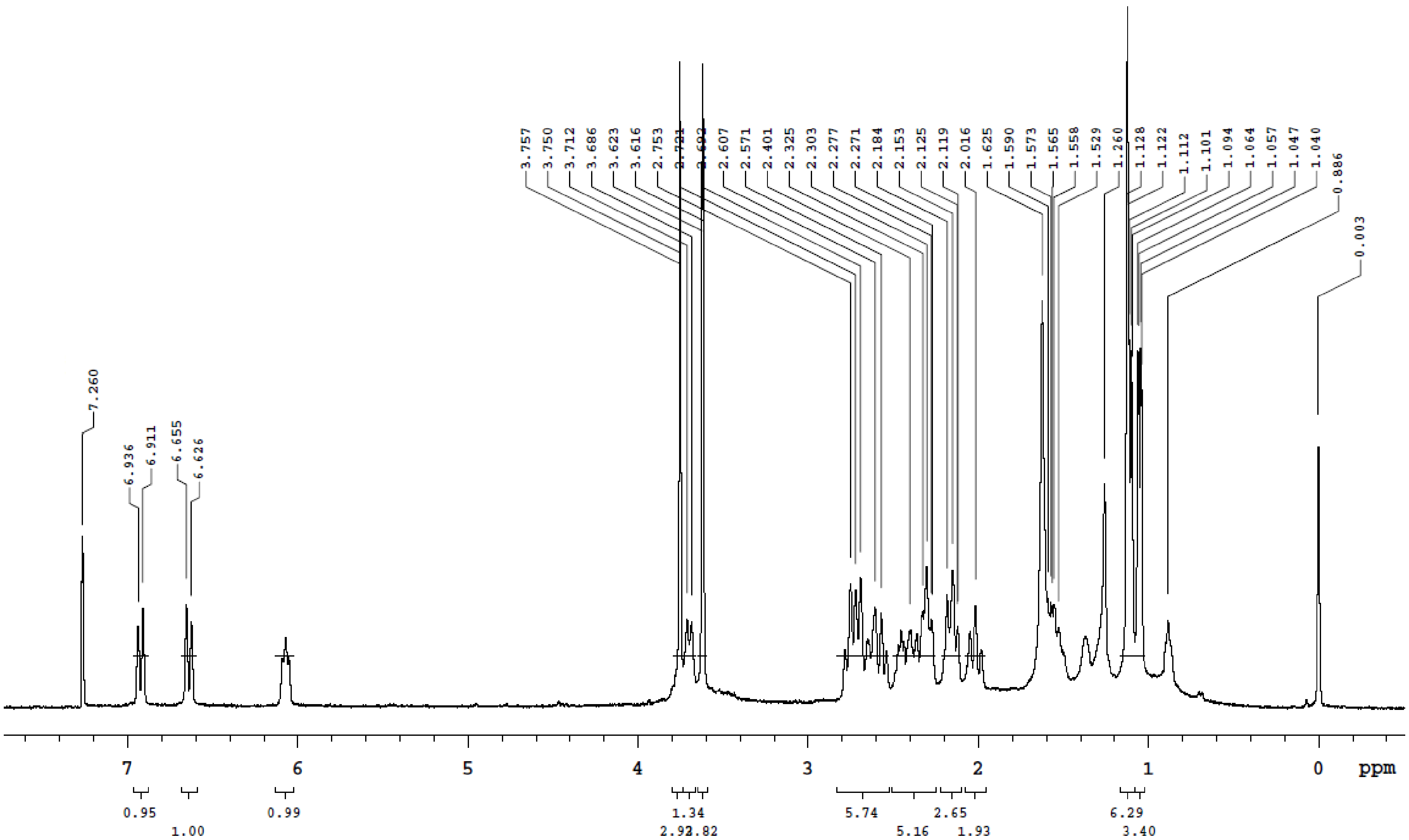

Figure S18. ^{1}H NMR spectrum of **3** in $CDCl_3$.

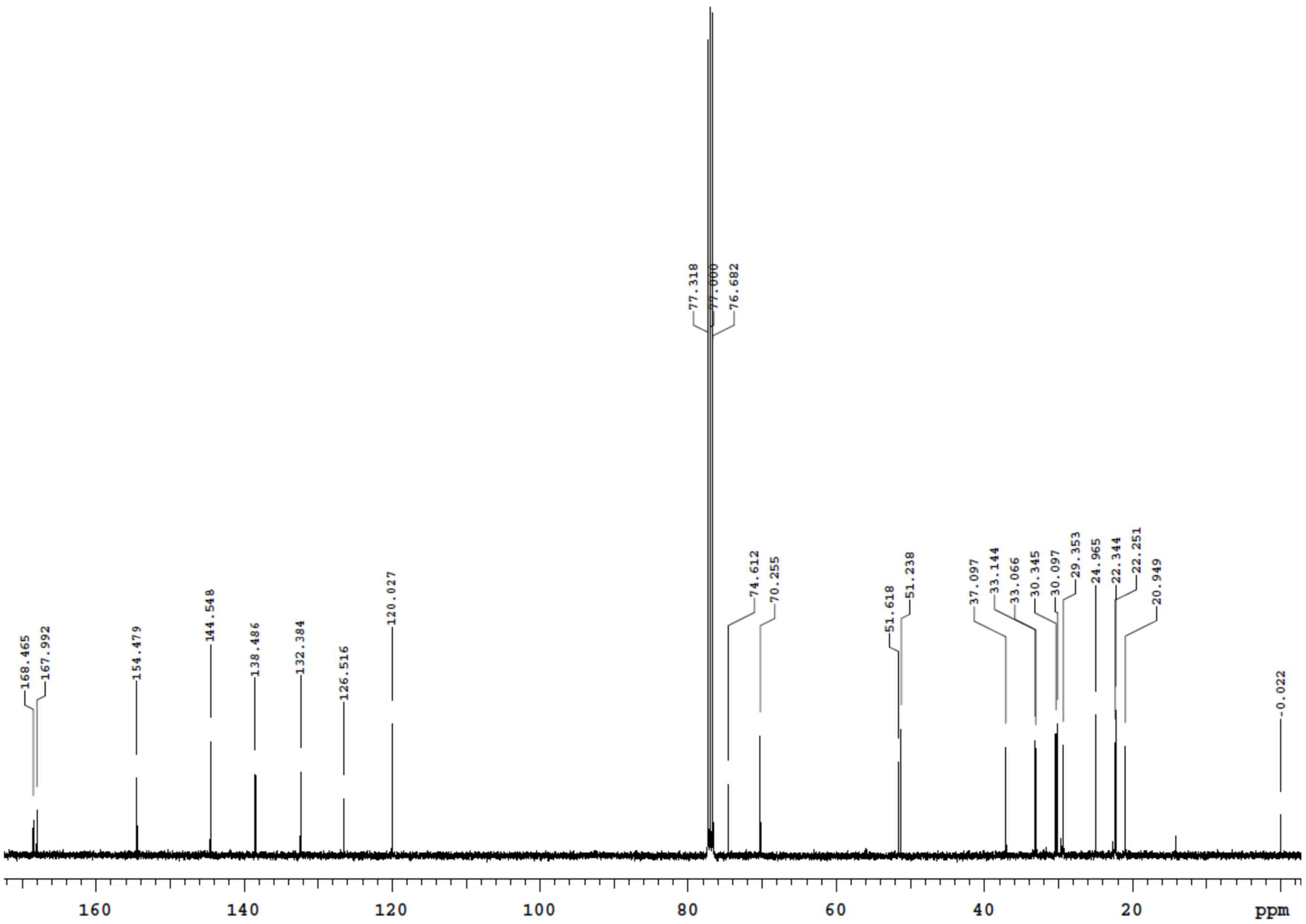

Figure S19. ^{13}C NMR spectrum of **3** in $CDCl_3$.

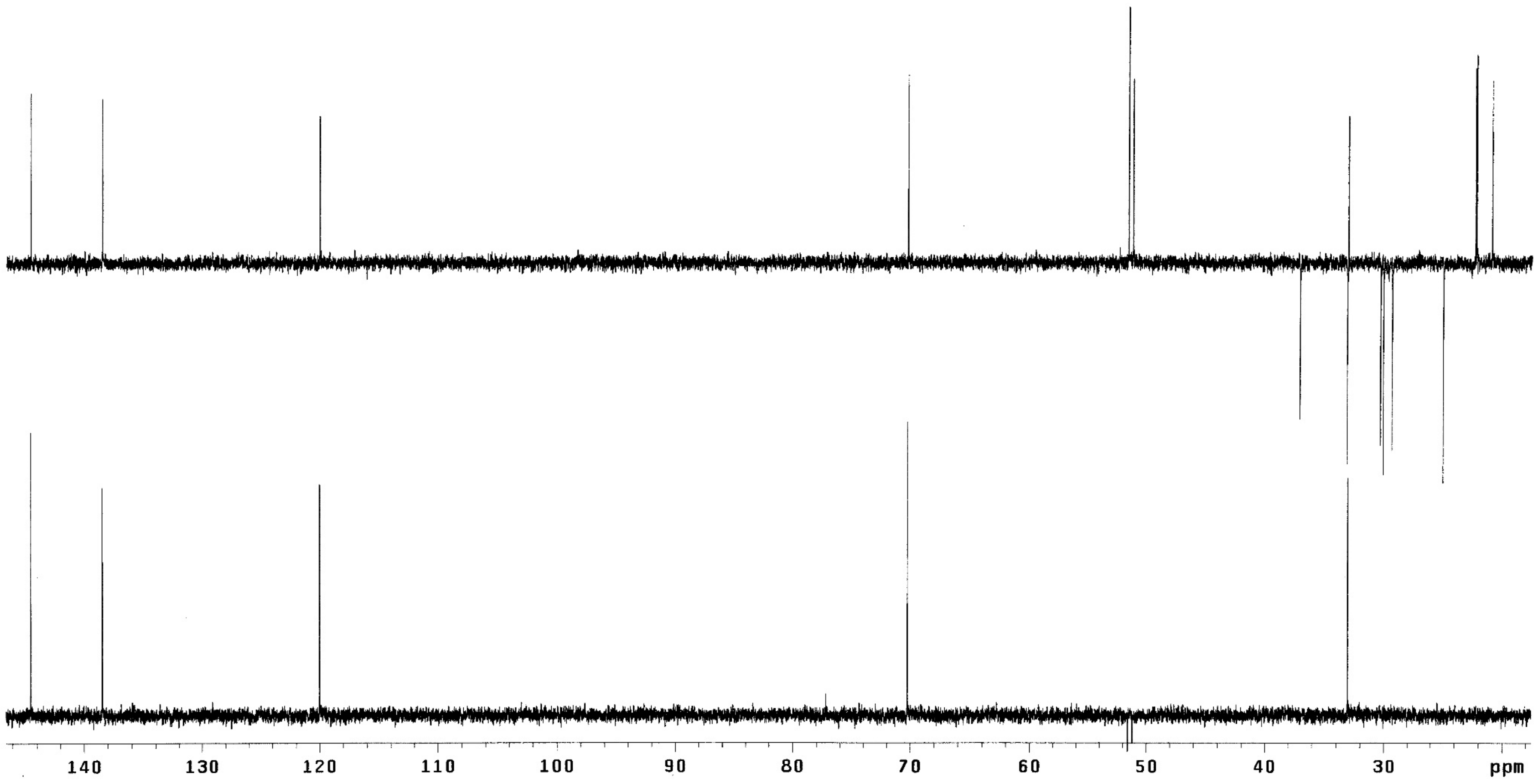

Figure S20. DEPT and ^{13}C NMR spectra of **3** in $CDCl_3$.

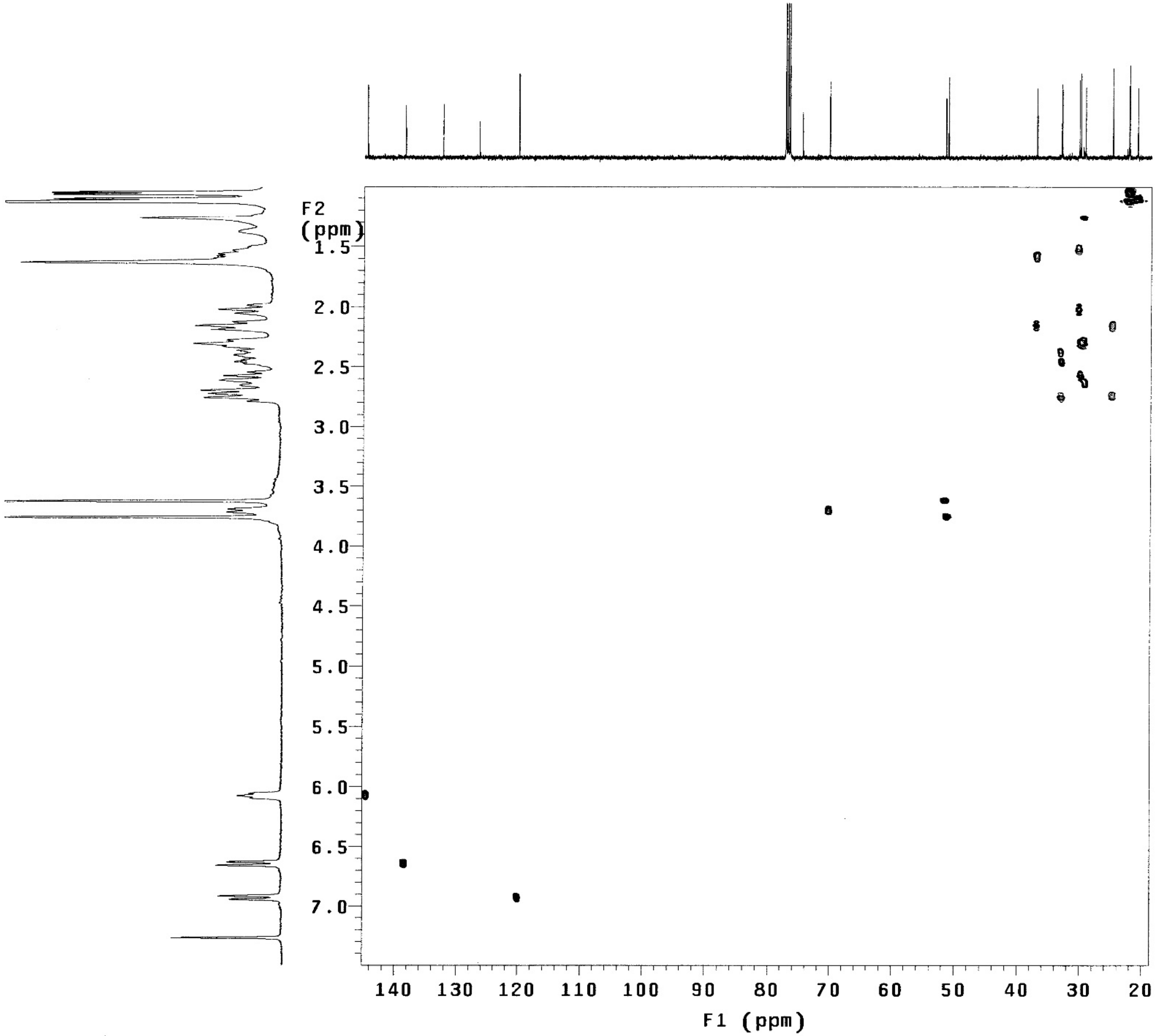

Figure S21. HSQC spectrum of **3** in $CDCl_3$.

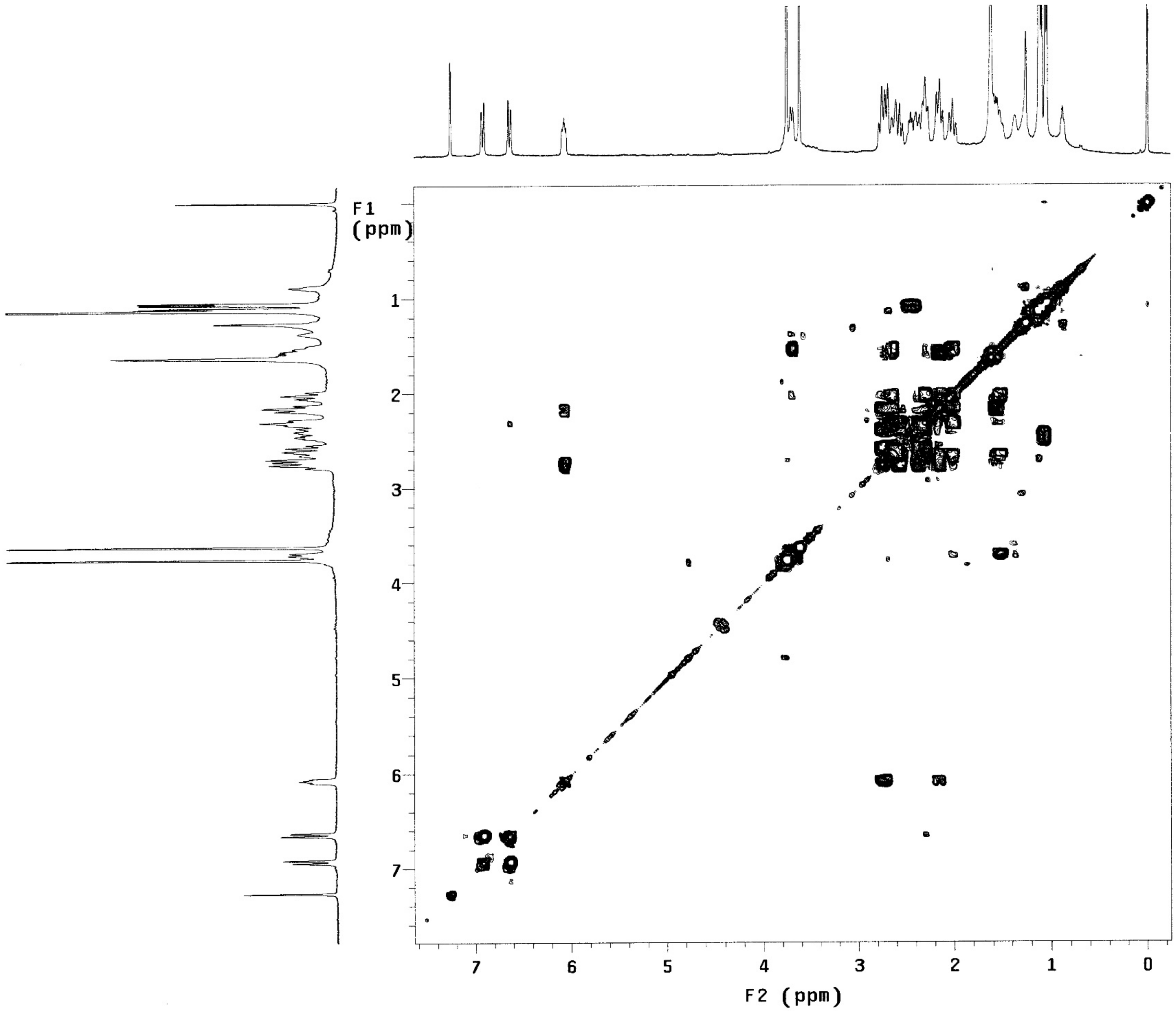

Figure S22. ^{1}H–^{1}H COSY spectrum of **3** in $CDCl_3$.

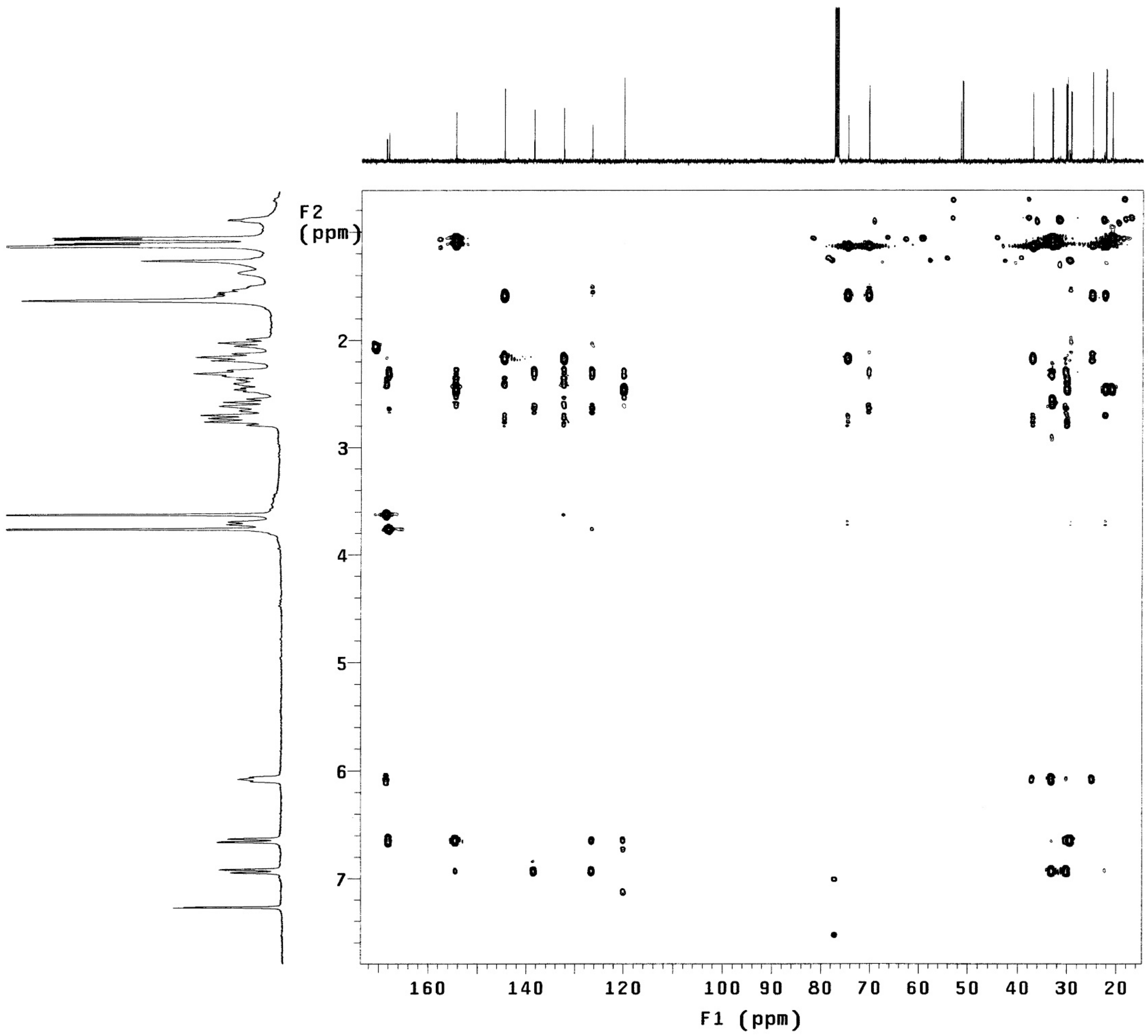

Figure S23. HMBC spectrum of **3** in $CDCl_3$.

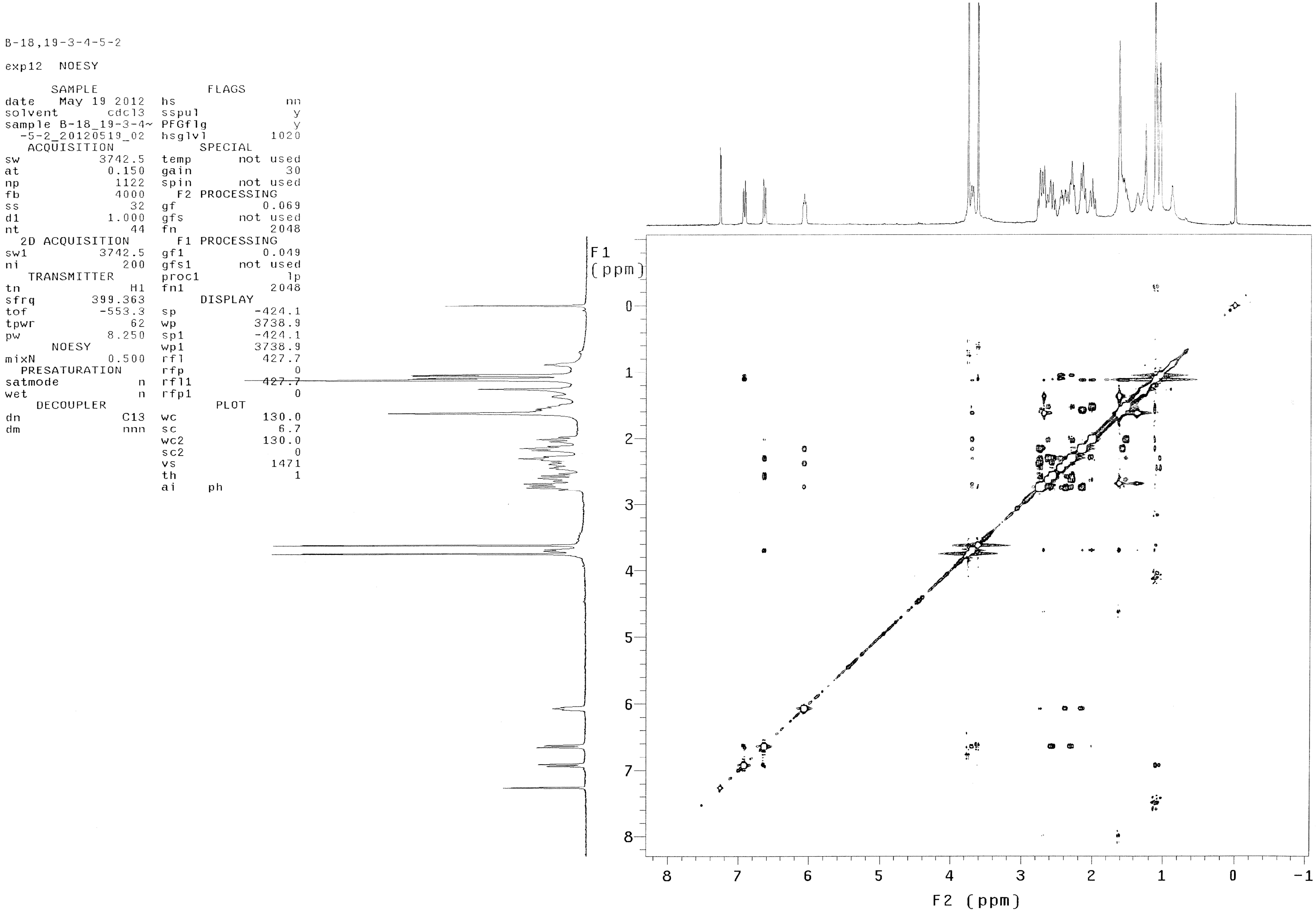

Figure S24. NOESY spectrum of **3** in $CDCl_3$.

Table S1. Low-energy conformers of 1*R*,2*S*,7*S*,8*R*-**1** for ECD calculations.

Conformer **1a** (97.39%)				Conformer **1b** (1.23%)			
C	2.904927	1.06325	0.256427	C	-2.41811	-1.72247	0.46399
C	2.878808	1.897627	-1.02391	C	-2.34163	-2.64425	-0.75443
C	4.284187	2.072305	-1.59999	C	-3.72198	-3.1858	-1.128
C	1.90126	1.388301	-2.08335	C	-1.62883	-2.04076	-1.96645
C	1.774619	1.145708	1.220206	C	-1.1983	-1.48513	1.289021
C	3.756667	-0.18802	0.332708	C	-3.53517	-0.70133	0.562309
O	2.990907	1.849364	1.435386	O	-2.20049	-2.41143	1.685449
C	0.572082	1.952948	0.943683	C	0.11259	-2.02721	0.883689
C	-0.67765	1.661168	1.33869	C	1.326235	-1.52047	1.157058
C	3.342914	-1.35379	-0.57191	C	-3.57955	0.379838	-0.52493
C	1.896328	-1.81916	-0.50627	C	-2.36223	1.282142	-0.6081
C	1.466101	-2.23491	0.873343	C	-2.17545	2.111395	0.629818
O	0.187197	-2.3821	1.26265	O	-1.05651	2.791542	0.918345
C	-1.02176	-2.54716	0.496306	C	0.211939	2.811558	0.240404
C	-0.78032	-3.29252	-0.8143	C	0.038231	3.223194	-1.22018
C	-0.14478	-2.49712	-1.93476	C	-0.46711	2.147748	-2.16348
C	1.206869	-1.94078	-1.65438	C	-1.6762	1.372668	-1.7621
O	2.303041	-2.38444	1.731948	O	-3.07601	2.195464	1.431042
C	-1.60665	-1.1368	0.270995	C	0.876596	1.42441	0.411568
C	-2.1181	-0.41621	1.521734	C	0.958879	0.98154	1.864133
C	-1.09638	0.499882	2.202889	C	1.662871	-0.3658	2.079072
C	-1.79087	2.580011	0.943422	C	2.449849	-2.23345	0.455508
O	-2.68988	-1.31573	-0.64964	O	2.203151	1.533058	-0.11407
C	-1.91347	-3.41583	1.378323	C	0.990643	3.901122	0.97074
C	-3.06848	-0.23955	-1.35711	C	2.638528	0.682797	-1.05792
C	-4.21773	-0.55613	-2.2673	C	4.073723	0.956701	-1.39651
O	-2.52653	0.831746	-1.25142	O	1.950648	-0.16992	-1.55921
O	-2.87878	2.572818	1.469319	O	2.336496	-3.26786	-0.15498
O	-1.47234	3.416162	-0.04313	O	3.618415	-1.59036	0.548474
C	-2.52195	4.249884	-0.51395	C	4.707305	-2.18329	-0.14942
H	2.537366	2.892275	-0.7023	H	-1.74847	-3.50343	-0.41083
H	4.268014	2.79918	-2.42434	H	-4.37245	-2.39233	-1.52661
H	4.674311	1.123892	-2.00038	H	-3.6264	-3.95808	-1.90436
H	4.985983	2.43731	-0.83561	H	-4.22196	-3.63593	-0.25772
H	2.263247	0.463641	-2.55199	H	-2.27996	-1.34355	-2.51044

H 1.794609 2.138284 -2.88046	H -1.35573 -2.84044 -2.67013
H 0.902427 1.193751 -1.66171	H -0.70804 -1.50614 -1.68291
H 1.649038 0.28688 1.888275	H -1.19081 -0.57711 1.899174
H 3.748578 -0.51719 1.379303	H -3.4666 -0.22506 1.55002
H 4.797939 0.079423 0.094844	H -4.49248 -1.24573 0.547358
H 0.722292 2.856725 0.351215	H 0.093462 -2.91272 0.245811
H 3.568282 -1.11386 -1.62073	H -3.74104 -0.07927 -1.50987
H 3.984448 -2.20843 -0.30585	H -4.45904 1.007501 -0.31942
H -0.15819 -4.17104 -0.57746	H 1.006781 3.589919 -1.58961
H -1.75159 -3.67407 -1.15591	H -0.645 4.086898 -1.22041
H -0.05522 -3.14125 -2.82356	H 0.321586 1.399865 -2.35893
H -0.80004 -1.67067 -2.26081	H -0.66921 2.598203 -3.14792
H 1.734989 -1.60918 -2.55366	H -2.05283 0.740348 -2.57149
H -0.83553 -0.51773 -0.21898	H 0.326894 0.67241 -0.17366
H -2.501 -1.14027 2.251404	H 1.516788 1.734481 2.43844
H -2.97564 0.198707 1.221772	H -0.05766 0.981089 2.277567
H -0.22431 -0.0821 2.525406	H 1.464146 -0.68599 3.114079
H -1.57011 0.908189 3.108664	H 2.743071 -0.19632 2.019705
H -2.94879 -3.37738 1.012577	H 0.931712 3.761547 2.056907
H -1.5632 -4.45621 1.34311	H 2.041721 3.885536 0.657457
H -1.88324 -3.08088 2.42143	H 0.55892 4.88128 0.727272
H -4.49369 0.336278 -2.83598	H 4.680757 0.863864 -0.48632
H -3.93187 -1.36973 -2.94701	H 4.415317 0.252852 -2.16097
H -5.06871 -0.90574 -1.6681	H 4.171011 1.988538 -1.75991
H -3.33678 3.630097 -0.91089	H 5.574702 -1.54044 0.026565
H -2.09223 4.867994 -1.30746	H 4.897986 -3.19578 0.228555
H -2.91012 4.881822 0.295249	H 4.480351 -2.24087 -1.22263
Conformer **1c** (1.11%)	
C 2.454268 1.69774 0.284003	
C 2.299563 2.850115 -0.70267	
C 3.63225 3.573899 -0.90309	
C 1.682769 2.409101 -2.0288	
C 1.302771 1.294628 1.139667	
C 3.583505 0.71435 -0.00823	
O 2.354501 2.111429 1.637173	
C -0.0467 1.868548 0.952755	
C -1.1941 1.305514 1.370296	

C	3.380681	-0.71485	0.479423	
C	2.255647	-1.49041	-0.18999	
C	1.894046	-2.71885	0.59055	
O	0.68348	-3.28729	0.533962	
C	-0.46547	-2.86396	-0.22636	
C	-0.12112	-2.89674	-1.7174	
C	0.667941	-1.70329	-2.23776	
C	1.765105	-1.14261	-1.39097	
O	2.702641	-3.21484	1.336877	
C	-0.95158	-1.46909	0.262309	
C	-0.79375	-1.25619	1.758817	
C	-1.3571	0.086679	2.256914	
C	-2.4818	1.920744	0.904711	
O	-2.33885	-1.35814	-0.07356	
C	-1.49335	-3.94642	0.087741	
C	-2.73992	-0.49124	-1.01771	
C	-4.21954	-0.58802	-1.24131	
O	-1.99515	0.265841	-1.58864	
O	-3.56854	1.427474	1.102263	
O	-2.32824	3.049368	0.21737	
C	-3.50953	3.606807	-0.34582	
H	1.61887	3.5679	-0.22369	
H	4.068613	3.865581	0.063476	
H	4.359788	2.942508	-1.43453	
H	3.483333	4.484487	-1.50039	
H	1.462719	3.285656	-2.65454	
H	0.743097	1.854778	-1.8741	
H	2.373471	1.769817	-2.60083	
H	1.321026	0.285476	1.554427	
H	4.488411	1.121372	0.468902	
H	3.794186	0.722141	-1.08752	
H	-0.12188	2.786145	0.36979	
H	3.206537	-0.71212	1.566857	
H	4.3182	-1.27787	0.352697	
H	-1.05493	-2.97477	-2.29382	
H	0.426309	-3.83675	-1.88658	
H	-0.02071	-0.86245	-2.42893	
H	1.091552	-1.94584	-3.22584	

H	2.216596	-0.25825	-1.84681	
H	-0.41799	-0.66989	-0.26347	
H	-1.29491	-2.06736	2.307432	
H	0.271807	-1.34314	2.008961	
H	-0.90284	0.302826	3.235572	
H	-2.43248	-0.03194	2.429919	
H	-1.70506	-3.98045	1.164788	
H	-2.42738	-3.76327	-0.45373	
H	-1.08328	-4.91865	-0.21623	
H	-4.50512	-1.63392	-1.41076	
H	-4.72325	-0.23237	-0.33279	
H	-4.50291	0.034177	-2.09552	
H	-3.95596	2.899503	-1.05783	
H	-3.20157	4.520996	-0.8607	
H	-4.24066	3.836028	0.439632	

Table S2. Low-energy conformers of **2** for ECD calculations.

Conformer **2a** (48.13%)				Conformer **2b** (45.14%)			
C	-2.28069	-1.67214	-0.34339	C	-2.69265	-0.9427	-0.3581
C	-3.50098	-0.77628	-0.5642	C	-3.6151	0.275237	-0.29273
C	-3.52106	-0.11075	-1.93876	C	-3.52398	1.173237	-1.52486
C	-4.77166	-1.60368	-0.34501	C	-5.05543	-0.19659	-0.07104
C	-1.09421	-1.56332	-1.24302	C	-1.60786	-1.00538	-1.3806
C	-2.11826	-2.26256	1.047537	C	-2.58777	-1.77432	0.908205
O	-2.06374	-2.59349	-1.3952	O	-2.82877	-1.716	-1.53648
C	0.242271	-2.04354	-0.84235	C	-0.39485	-1.81895	-1.17441
C	1.42418	-1.47965	-1.13551	C	0.855001	-1.46604	-1.5167
C	-2.16524	-1.23534	2.185468	C	-2.18399	-0.98688	2.158597
C	-1.24086	-0.04084	2.023594	C	-0.94891	-0.11376	2.02014
C	0.216485	-0.40504	2.141334	C	0.312723	-0.90869	1.804493
O	1.241927	0.430752	1.89362	O	1.48312	-0.39464	1.385763
C	1.238366	1.756047	1.332368	C	1.877043	0.974256	1.171383
C	0.262742	2.683096	2.054956	C	1.380361	1.889049	2.291173
C	-1.20496	2.532813	1.720783	C	-0.07401	2.307415	2.242232
C	-1.79472	1.177282	1.883233	C	-1.0792	1.212	2.205117
O	0.511438	-1.51916	2.502836	O	0.288872	-2.10205	1.988874
C	0.932545	1.583911	-0.17084	C	1.353491	1.390109	-0.2233
C	2.072335	0.940592	-0.97273	C	2.083486	0.767164	-1.42427
C	1.644016	-0.20373	-1.90182	C	1.25181	-0.21484	-2.25167
C	2.626924	-2.21265	-0.61585	C	1.988206	-2.36284	-1.13101
O	0.657193	2.895537	-0.67836	O	1.482379	2.816971	-0.27833
C	2.646857	2.287306	1.583876	C	3.401313	0.935497	1.237187
C	-0.1726	2.998146	-1.72653	C	0.700995	3.451941	-1.16541
C	-0.37898	4.429504	-2.12475	C	0.907619	4.937057	-1.11984
O	-0.68444	2.041492	-2.25165	O	-0.07063	2.868838	-1.8842
O	2.598504	-3.22835	0.034733	O	3.150951	-2.08857	-1.32303
O	3.770123	-1.61721	-0.96569	O	1.607852	-3.49627	-0.54601
C	4.966852	-2.24064	-0.51486	C	2.647059	-4.33337	-0.05936
H	-3.46689	0.017118	0.196021	H	-3.30254	0.867445	0.581011
H	-2.71242	0.623937	-2.05356	H	-2.54806	1.673335	-1.59968
H	-4.47611	0.412537	-2.08542	H	-4.29727	1.952295	-1.47792
H	-3.42386	-0.86907	-2.73071	H	-3.69003	0.58519	-2.44067
H	-4.78722	-2.45975	-1.03724	H	-5.36173	-0.8637	-0.89154

H	-5.66434 -0.99224 -0.53677			H	-5.74178 0.661289 -0.05207		
H	-4.846 -1.9906 0.681159			H	-5.17432 -0.74055 0.876514		
H	-1.12224 -0.78859 -2.01466			H	-1.50684 -0.14574 -2.0464		
H	-1.17888 -2.82217 1.104499			H	-1.88404 -2.59808 0.751509		
H	-2.92469 -2.99563 1.204314			H	-3.56791 -2.24156 1.090163		
H	0.269112 -2.97856 -0.27921			H	-0.53262 -2.77798 -0.67594		
H	-3.19055 -0.85595 2.298817			H	-3.01659 -0.34479 2.478544		
H	-1.91236 -1.75729 3.119048			H	-2.01147 -1.71011 2.968638		
H	0.567522 3.716222 1.8401			H	2.005945 2.791528 2.281214		
H	0.419916 2.522418 3.133335			H	1.589366 1.367396 3.239082		
H	-1.78659 3.226436 2.347594			H	-0.29616 2.925769 3.126562		
H	-1.41275 2.860174 0.687404			H	-0.26639 2.975933 1.386967		
H	-2.88985 1.187124 1.880987			H	-2.10254 1.55739 2.388702		
H	0.012201 0.98194 -0.26292			H	0.278193 1.143468 -0.26848		
H	2.556579 1.740425 -1.55205			H	2.389654 1.591724 -2.08313		
H	2.837633 0.549051 -0.29244			H	3.005577 0.269274 -1.10989		
H	2.443874 -0.37356 -2.63509			H	1.857272 -0.51258 -3.12149		
H	0.738157 0.081241 -2.45058			H	0.364354 0.306478 -2.63178		
H	2.755335 2.556462 2.643133			H	3.722713 0.859017 2.28464		
H	3.409749 1.539556 1.342202			H	3.79388 0.067724 0.694562		
H	2.813947 3.185339 0.973252			H	3.816741 1.856832 0.805585		
H	-1.02543 4.477157 -3.00545			H	0.319254 5.414663 -1.90832		
H	-0.83592 4.975299 -1.28803			H	0.595486 5.314499 -0.13628		
H	0.592632 4.895046 -2.33413			H	1.974166 5.165017 -1.24077		
H	5.041206 -3.25928 -0.91683			H	2.15812 -5.21213 0.37055		
H	4.981981 -2.28854 0.581681			H	3.227353 -3.80719 0.710527		
H	5.79206 -1.62414 -0.88204			H	3.319723 -4.62974 -0.87444		
Conformer **2c** (1.64%)				Conformer **2d** (1.59%)			
C	3.049806	-0.22021	-0.52958	C	0.269877	2.470082	-0.67802
C	3.451032	-1.66357	-0.82564	C	1.780452	2.706742	-0.72853
C	4.814548	-1.99527	-0.22092	C	2.442519	2.117326	-1.97055
C	2.393707	-2.69873	-0.4434	C	2.048103	4.214589	-0.6551
C	2.146449	0.42364	-1.52178	C	-0.37989	1.421017	-1.53236
C	3.176236	0.335643	0.877254	C	-0.43745	2.981751	0.569074
O	3.541528	0.686102	-1.50649	O	-0.39532	2.798641	-1.88298
C	1.277861	1.564002	-1.17076	C	-1.70471	0.874436	-1.17249
C	-0.01906	1.703655	-1.48861	C	-2.12216	-0.39968	-1.23404

C	2.392344	-0.34486	2.003062	C	0.235403	2.653635	1.90742
C	0.881704	-0.44901	1.871026	C	0.638123	1.215483	2.146439
C	0.19583	0.88083	1.712074	C	-0.43451	0.189587	2.001753
O	-1.04402	1.038937	1.213048	O	-0.12683	-1.12555	2.077984
C	-2.08178	0.061361	1.003354	C	0.926879	-1.84588	1.403479
C	-2.15359	-0.91409	2.180754	C	2.341992	-1.31343	1.701558
C	-1.13172	-2.03308	2.198043	C	2.375658	-0.45562	2.96457
C	0.299924	-1.63846	2.102667	C	1.843158	0.9135	2.660451
O	0.792386	1.887604	2.008352	O	-1.59861	0.498691	1.940006
C	-1.82999	-0.6615	-0.34364	C	0.636928	-1.75668	-0.10534
C	-2.07193	0.147751	-1.62568	C	-0.6999	-2.35237	-0.53773
C	-0.82773	0.731045	-2.3032	C	-1.3485	-1.59873	-1.71202
C	-0.753	2.913825	-1.01014	C	-3.46396	-0.73447	-0.66285
O	-2.74109	-1.77051	-0.33281	O	1.720634	-2.4598	-0.72807
C	-3.36477	0.885585	0.982042	C	0.785488	-3.26801	1.927712
C	-2.41242	-2.8344	-1.08223	C	2.201945	-2.04631	-1.90551
C	-3.44693	-3.9166	-0.98217	C	3.369298	-2.88178	-2.34106
O	-1.40118	-2.88392	-1.73433	O	1.746632	-1.11155	-2.51555
O	-1.9185	3.130364	-1.25653	O	-3.85012	-1.86999	-0.50996
O	-0.01209	3.738374	-0.27607	O	-4.19792	0.323799	-0.31958
C	-0.6948	4.817352	0.343299	C	-5.45215	0.046069	0.288752
H	3.556783	-1.68967	-1.9223	H	2.216911	2.225255	0.16136
H	5.56662	-1.24497	-0.50653	H	2.407716	1.021028	-1.98096
H	4.766614	-2.03403	0.878408	H	3.496497	2.425465	-2.01371
H	5.160208	-2.97768	-0.57279	H	1.941535	2.48765	-2.87802
H	2.648724	-3.67022	-0.89104	H	1.55621	4.722601	-1.49919
H	1.39137	-2.41409	-0.79975	H	3.126896	4.413777	-0.71934
H	2.342977	-2.84509	0.643381	H	1.679741	4.665561	0.276802
H	1.824049	-0.21157	-2.35379	H	0.27217	0.789599	-2.14546
H	2.917829	1.400087	0.852101	H	-1.46626	2.610981	0.591561
H	4.244616	0.29001	1.143119	H	-0.50412	4.077542	0.482168
H	1.749376	2.3537	-0.5849	H	-2.41441	1.610346	-0.79509
H	2.784333	-1.35645	2.177239	H	-0.46782	2.951916	2.701323
H	2.604468	0.228871	2.918766	H	1.134832	3.271972	2.041223
H	-3.15846	-1.3562	2.189646	H	2.714246	-0.71403	0.856378
H	-2.06521	-0.30724	3.096337	H	3.019463	-2.17249	1.798769
H	-1.33066	-2.764	1.396562	H	1.785968	-0.93797	3.761257
H	-1.25135	-2.61229	3.127875	H	3.4051	-0.36973	3.331127

H	0.988209	-2.4706	2.282671	H	2.524016	1.753525	2.835162
H	-0.80093	-1.05776	-0.34134	H	0.700739	-0.69122	-0.39143
H	-2.52467	-0.54279	-2.3502	H	-0.52752	-3.40701	-0.79895
H	-2.80724	0.939491	-1.4507	H	-1.396	-2.34257	0.313196
H	-1.16912	1.248078	-3.21415	H	-2.05265	-2.27279	-2.2172
H	-0.18937	-0.10127	-2.6272	H	-0.5837	-1.29897	-2.43898
H	-4.19229	0.273388	0.597481	H	1.450662	-3.93514	1.364661
H	-3.60829	1.209724	2.00304	H	1.067638	-3.29861	2.989667
H	-3.24241	1.775611	0.353106	H	-0.24971	-3.61898	1.832423
H	-3.16422	-4.7565	-1.62299	H	3.087397	-3.94222	-2.33398
H	-4.42411	-3.51653	-1.28213	H	3.689048	-2.57436	-3.34057
H	-3.52883	-4.24616	0.062421	H	4.190706	-2.74984	-1.62371
H	-1.45755	4.432294	1.034081	H	-6.08866	-0.53749	-0.38905
H	0.06106	5.383924	0.894605	H	-5.91165	1.015319	0.50206
H	-1.17933	5.454816	-0.4074	H	-5.30825	-0.52051	1.218188
Conformer **2e** (1.55%)							
C	-2.19276	-1.96623	-0.248				
C	-3.56504	-1.35034	-0.52164				
C	-4.67741	-2.32604	-0.13797				
C	-3.75247	0.018943	0.123858				
C	-1.15273	-1.67721	-1.28149				
C	-1.92433	-2.39784	1.193904				
O	-1.85458	-2.91177	-1.24706				
C	0.303264	-1.8084	-1.08934				
C	1.255718	-0.97173	-1.53604				
C	-0.55419	-2.13737	1.829503				
C	-0.12789	-0.67482	1.865053				
C	1.354896	-0.48691	1.68472				
O	1.924	0.653169	1.257785				
C	1.316643	1.937568	1.028856				
C	0.450664	2.355031	2.218283				
C	-0.93575	1.748747	2.280722				
C	-1.03519	0.26651	2.170547				
O	2.10115	-1.41032	1.905114				
C	0.53374	1.843267	-0.29541				
C	1.410482	1.567046	-1.52501				
C	1.029606	0.303009	-2.30453				

C	2.687094	-1.31132	-1.25698	
O	-0.15533	3.093889	-0.41865	
C	2.492468	2.904375	0.933461	
C	-1.23035	3.118396	-1.21894	
C	-1.88905	4.466103	-1.22038	
O	-1.60692	2.1508	-1.8315	
O	3.610193	-0.57678	-1.52204	
O	2.856194	-2.5029	-0.68804	
C	4.164772	-2.7957	-0.21895	
H	-3.60439	-1.21613	-1.61455	
H	-4.51704	-3.30396	-0.61451	
H	-4.72617	-2.47441	0.951537	
H	-5.65382	-1.94024	-0.46408	
H	-4.67742	0.483878	-0.24678	
H	-2.915	0.691262	-0.11885	
H	-3.84494	-0.05617	1.217914	
H	-1.47343	-0.99537	-2.07818	
H	-2.70205	-1.94358	1.824366	
H	-2.12101	-3.48052	1.235334	
H	0.620911	-2.72065	-0.58549	
H	-0.58896	-2.52187	2.861761	
H	0.226954	-2.72691	1.341402	
H	0.354438	3.449025	2.199608	
H	1.021299	2.102487	3.126223	
H	-1.57545	2.173398	1.485778	
H	-1.42566	2.055081	3.218391	
H	-2.04483	-0.10346	2.364786	
H	-0.22348	1.04861	-0.19888	
H	1.339762	2.437665	-2.1933	
H	2.462665	1.479907	-1.23356	
H	1.652459	0.266509	-3.21124	
H	-0.01884	0.378661	-2.61739	
H	2.14429	3.869208	0.541123	
H	2.91944	3.061626	1.933237	
H	3.281782	2.511145	0.283011	
H	-2.67609	4.489838	-1.97936	
H	-1.14204	5.246708	-1.40986	
H	-2.32229	4.651592	-0.22754	

H	4.438126	-2.08551	0.572436	
H	4.126541	-3.81211	0.183508	
H	4.893399	-2.73506	-1.03723	

Table S3. Conformers of 1*R**,2*S**,7*S**,8*S**-**1** for *J*-DP4 calculation and the dihedral angles of selected protons at MMFF94 level (the selected conformers were marked in yellow).

conformer	MMFF94 rel. energy (kcal/mol)	Dihedral angles at MMFF94 level H-2/H-3[a]	H-7/H-6 *pro-R*[b]	H-7/H-6 *pro-S*[b]
1	0	-114.8	166.8	-78.8
2	0.0503	-115.3	163.8	-81.6
3	0.3797	-115.6	166.3	-79.3
4	0.7148	-116.2	-49.3	64.1
5	0.8867	-114.1	-49.1	64.3
6	1.3223	-125.9	167.2	-79.6
7	1.362	-118.3	176.9	-70.0
8	1.3671	-125.1	168.0	-78.7
9	1.5598	-118.3	178.1	-68.8
10	1.6966	-131.8	-46.6	68.2
11	1.8095	-133.4	-46.6	68.0
12	1.9409	-165.7	-38.8	75.8
13	2.0214	-143.5	-41.7	73.1
14	2.0233	-149.1	-40.1	75.0
15	2.1268	-162.0	-37.8	77.0
16	2.4117	-161.3	-58.1	55.0
17	2.4494	-163.4	-58.2	55.0
18	2.6174	-144.3	-42.1	72.8
19	2.668	-162.6	-58.1	55.1
20	2.6981	-123.9	-175.4	-63.2
21	2.7009	-114.0	177.5	-69.2
22	2.8633	-160.6	-58.3	54.9
23	2.879	-114.5	178.2	-68.6
24	2.9183	-154.6	-174.5	-63.5
25	2.9642	-113.8	162.8	-82.4
26	2.9754	-112.8	160.2	-83.6
27	3.0523	-125.8	-176.4	-64.2
28	3.1159	-126.4	-177.0	-64.9
29	3.1468	-179.8	-168.6	-57.3
30	3.241	-134.6	-61.7	53.0
31	3.2605	-166.8	-172.2	-61.4
32	3.2696	-134.3	-63.0	51.7
33	3.2984	-163.4	-172.8	-61.6
34	3.5209	176.8	-169.5	-58.5
35	3.5798	-135.1	-57.8	56.8
36	3.6432	-115.1	171.7	-73.5
37	3.673	-115.3	-49.3	64.1
38	3.688	-157.5	-173.8	-63.2
39	3.7558	-113.2	159.4	-84.3
40	3.7573	174.8	115.5	-127.8
41	3.7666	-117.4	69.0	-170.7
42	3.8081	-133.1	-60.1	54.5

43	3.8093	-135.8	-62.1	52.5
44	3.834	-113.4	-49.1	64.3
45	3.8767	-115.5	71.1	-168.5
46	3.9137	-111.8	-42.6	71.3
47	3.945	-140.8	-42.5	72.2
48	3.974	-135.8	-61.6	53.1
49	4.049	-135.2	-59.0	55.4
50	4.1323	-154.8	-37.9	77.2
51	4.1586	-136.8	-57.9	56.7
52	4.1639	-124.2	162.5	-82.5
53	4.187	-110.3	-43.1	70.8
54	4.2541	172.7	125.5	-118.8
55	4.3108	-154.9	-37.5	77.5
56	4.3579	-178.5	109.8	-133.1
57	4.3682	-115.2	-176.0	-63.2
58	4.4068	-115.3	171.3	-73.9
59	4.4143	-143.3	-173.6	-62.5
60	4.5492	-158.4	-57.7	55.4
61	4.5578	-121.2	167.6	-79.1
62	4.561	-162.9	-170.7	-59.4
63	4.5633	121.4	167	-79.6
64	4.5845	-142.4	-42.6	72.1
65	4.6488	-160.4	-57.8	55.4
66	4.6702	-128.5	-46.6	68.1
67	4.6751	172.3	132	111.8
68	4.7162	-108.1	-49.3	64.5
69	4.7535	168.7	130.1	-114.4
70	4.762	-116.9	-177.2	-64.5
71	4.7808	-117	-178.1	-65.5
72	4.832	-124.9	162.1	-82.9
73	4.8534	-159.8	-57.6	55.6
74	4.8746	-146.1	-172.8	-62
75	4.9269	-112.7	172.3	-72.8
76	4.9917	-157.8	-57.8	55.3
77	5.0177	-111.3	-49.7	64.2
78	5.0382	-174.7	-38	76.7
79	5.0608	-102	179.3	-67.9
80	5.1246	-133.3	-58	56.7
81	5.2184	172.3	102.6	-139.6

[a.] Conformers showing dihedral angles over 140° were selected and the vinyl $^3J_{H,H}$ values can be estimated using the modified Karplus Equation by Garbisch (Garbisch, E. W., Jr. *J. Am. Chem. Soc.* **1964**, *86*, 5561–5564). [b.] Small $^3J_{H,H}$ (< 4 Hz) values were selected based on dihedral angles in the range 50–120° and large $^3J_{H,H}$ (> 8 Hz) values were in the ranges 0–30° and /or 140–180°.

Table S4. Conformers of 1*R**,2*S**,7*R**,8*S**-**1** for *J*-DP4 calculation and the dihedral angles of selected protons at MMFF94 level (the selected conformers were marked in yellow).

conformer	MMFF94 rel. energy (kcal/mol)	Dihedral angles at MMFF94 level H-2/H-3[a]	H-7/H-6 *pro-R*[b]	H-7/H-6 *pro-S*[b]
1	0	-113.9	80.9	-166.1
2	0.9814	-155.2	79.4	-167.9
3	1.4706	-113.2	92.9	-152.3
4	1.4913	171.1	80.8	-166.2
5	1.5453	173.6	79.8	-167.1
6	1.8883	-114.7	-70.7	44.2
7	2.053	-135.1	83.8	-161.0
8	2.0937	-114.4	-70.7	44.2
9	2.1401	-114.3	-72.0	42.7
10	2.1944	173.7	83.2	-163.4
11	2.3006	176.7	82.3	-164.1
12	2.3032	-156.2	170.4	-71.9
13	2.351	-115.2	90.4	-155.1
14	2.4791	-125.4	-69.0	45.0
15	2.4832	-119.3	67.3	-179.8
16	2.6149	-125.5	-69.8	44.0
17	2.6744	-115.2	-71.2	43.6
18	2.6946	-164.5	63.9	175.5
19	2.7274	-136.7	83.9	-160.9
20	2.7659	-117.3	67.9	-179.2
21	2.7673	-162.6	63.7	175.2
22	2.9523	-156.3	170.1	-72.2
23	2.9854	-117.4	-62.9	51.6
24	2.9946	-125.7	-68.7	45.1
25	3.0161	-113.6	80.8	-166.3
26	3.0242	-162.4	62.7	174.0
27	3.086	-164.4	62.8	174.1
28	3.216	-140.0	89.5	-154.6
29	3.3279	-117.1	-64.0	50.3
30	3.3957	-118.3	67.9	-179.2
31	3.5471	-150.9	171.9	-70.2
32	3.9464	-147.5	161.6	-80.1
33	4.0261	-154.3	89.6	-154.8
34	4.034	-114.3	79.4	-167.9
35	4.1515	173.4	81.7	-165.2
36	4.1673	177.1	81.8	-164.8
37	4.2281	-127.2	-58.3	55.7
38	4.2555	-151.2	171.9	-70.3
39	4.2844	-114.0	-62.6	52.1
40	4.2935	-126.8	-58.3	55.7
41	4.2945	-152.6	-54.3	58.2
42	4.4039	-126.5	-58.9	55
43	4.4687	-107.1	63.4	176.3

44	4.4815	-128.4	170.6	-71.1
45	4.6474	-147.2	161.4	-80.4
46	4.6558	-113.4	-63.6	50.8
47	4.6958	-156.4	89.6	-154.8
48	4.7279	-126.1	-57.7	56.2
49	4.739	-161.3	92.4	-152
50	4.7794	-113.4	-71.3	43.6
51	4.8287	-133.4	58.2	170.6
52	4.9241	-150.6	-53.9	58.6
53	4.9568	-113.1	-71.3	43.5
54	4.9728	-161.3	64.2	175.9
55	4.9746	-113.9	82.3	-163.2
56	4.9922	-159.6	64.0	175.6
57	5.0376	-113.0	-72.6	42.1
58	5.0705	-150.1	-53.3	59.3
59	5.1581	-107.8	63.5	176.4
60	5.2551	-159.2	63.1	174.4
61	5.3019	-161.4	-51.5	61.4
62	5.3488	-128.4	171.3	-70.5
63	5.3763	-161.1	63.2	174.6
64	5.4268	-134.5	57.2	169.6
65	5.4512	-134.6	58.3	170.6

[a.] Conformers showing dihedral angles over 140° were selected and the vinyl $^3J_{H,H}$ values can be estimated using the modified Karplus Equation by Garbisch (Garbisch, E. W., Jr. *J. Am. Chem. Soc.* **1964**, *86*, 5561–5564). [b.] Small $^3J_{H,H}$ (< 4 Hz) values were selected based on dihedral angles in the range 50–120° and large $^3J_{H,H}$ (> 8 Hz) values were in the ranges 0–30° and /or 140–180°.

Table S5. Conformers of 1*R**,2*S**,7*S**,8*R**-**1** for *J*-DP4 calculation and the dihedral angles of selected protons at MMFF94 level (the selected conformers were marked in yellow).

conformer	MMFF94 rel. energy (kcal/mol)	Dihedral angles at MMFF94 level H-2/H-3[a]	H-7/H-6 *pro-R*[b]	H-7/H-6 *pro-S*[b]
1	0	-176.9	176.3	-71.5
2	1.019	-179.4	177.0	-71.0
3	2.7493	-169.4	176.9	-71.0
4	3.887	-154.9	-38.1	77.0
5	3.8955	-172.0	178.3	-69.8
6	3.9172	-161.7	177.8	-69.9
7	4.0306	-155.7	-37.7	77.6
8	4.0672	-158.3	-36.7	78.4
9	4.43	-157.0	-38.0	77.1
10	4.643	-165.4	171.1	-70.2
11	4.832	-177.7	171.0	-75.0
12	5.1697	-174.8	170.9	-75.4
13	5.6161	-179.3	67.2	-177.4
14	6.2459	-176	66.5	-177.7

[a] Conformers showing dihedral angles over 140° were selected and the vinyl $^3J_{H,H}$ values can be estimated using the modified Karplus Equation by Garbisch (Garbisch, E. W., Jr. *J. Am. Chem. Soc.* **1964**, *86*, 5561–5564). [b] Small $^3J_{H,H}$ (< 4 Hz) values were selected based on dihedral angles in the range 50–120° and large $^3J_{H,H}$ (> 8 Hz) values were in the ranges 0–30° and /or 140–180°.

Table S6. Conformers of 1*R**,2*S**,7*R**,8*R**-**1** for *J*-DP4 calculation and the dihedral angles of selected protons at MMFF94 level (the selected conformers were marked in yellow).

conformer	MMFF94 rel. energy (kcal/mol)	Dihedral angles at MMFF94 level		
		H-2/H-3[a]	H-7/H-6 *pro-R*[b]	H-7/H-6 *pro-S*[b]
1	0	-173.3	-56.8	56.4
2	1.1514	-168.8	-56.3	56.9
3	2.1387	-154.4	87.6	-156.8
4	2.2075	-157.8	91.5	-152.5
5	2.4439	-157.5	92.5	-151.5
6	2.4895	-168.7	-56.7	56.5
7	3.4132	-164.8	-56.1	57.1
8	4.2175	-154.3	91.6	-152.3
9	4.5219	-147.9	82.5	-162.7
10	4.5525	-149.4	85.0	-159.9
11	4.5605	-154.1	88.6	-156.5
12	4.595	-152.6	87.3	-157.1
13	4.748	-152.8	88.5	-156.6
14	4.9092	-148.1	82.5	-162.7
15	5.049	-150.8	87.6	-157.4
16	5.2953	-124.6	-47.9	67.1
17	5.3583	-124.1	-49.2	65.7

[a] Conformers showing dihedral angles over 140° were selected and the vinyl $^3J_{H,H}$ values can be estimated using the modified Karplus Equation by Garbisch (Garbisch, E. W., Jr. *J. Am. Chem. Soc.* **1964**, *86*, 5561–5564). [b] Small $^3J_{H,H}$ (< 4 Hz) values were selected based on dihedral angles in the range 50–120° and large $^3J_{H,H}$ (> 8 Hz) values were in the ranges 0–30° and /or 140–180°.

Table S7. Conformers of 1*R**,2*R**,7*R**,8*R**-**2** for *J*-DP4 calculation and the dihedral angles of selected protons at MMFF94 level (the selected conformers were marked in yellow).

conformer	MMFF94 rel. energy (kcal/mol)	Dihedral angles at MMFF94 level H-2/H-3[a]	H-7/H-6 *pro-R*[b]	H-7/H-6 *pro-S*[b]
1	0	72	150.2	-94.3
2	0.1034	87.5	-56.6	56
3	0.3151	75.6	153.3	-91.6
4	0.4103	82.9	-53.9	58.6
5	0.6114	71.1	154.9	-90
6	0.8517	71.5	149.1	-95.3
7	0.8883	69.8	154.1	-90.7
8	1.0333	-149.8	-44.8	67.8
9	1.1472	75	153.3	-91.6
10	1.2774	73.5	156.7	-88.5
11	1.2915	-147.5	141.9	-102.4
12	2.1929	-147.1	145.8	-98.8
13	2.3801	76.5	-50.6	61.9
14	2.5059	-148.3	141	-103.3
15	2.5459	87.1	-58	54.6
16	2.5487	91.5	92.8	-149.3
17	2.5749	-147.6	-47.2	65.4
18	2.5866	72.8	150.7	-94.2
19	2.6492	94.5	-173.5	-62.6
20	2.7529	92.3	92.3	-149.7
21	2.8419	-150.1	-44	68.6
22	2.9572	-144.5	144.5	-100.1
23	3.1574	93.4	-173.1	-62.3
24	3.2239	-148.3	144.7	-99.9
25	3.314	71.5	85.8	-156.9
26	3.3628	72.3	151.6	-93.4
27	3.5767	91.8	-57.5	55.2
28	3.5775	70.6	86.2	-156.6
29	3.7057	-144.4	148.7	-96.3
30	3.792	99.1	119.4	-124.5
31	3.811	99.7	119.4	-124.5
32	3.8688	91.3	91.8	-150.3
33	3.9242	92.9	114	-129.6
34	4.0816	92.2	91.4	-150.7
35	4.0923	-157.5	134.2	-110
36	4.1074	93.9	113.2	-130.3
37	4.1878	88.2	76.9	-166
38	4.2035	-134.1	-163.7	-52.9
39	4.2136	-135.3	-165.4	-54.7
40	4.2488	73.3	65.7	-179.4
41	4.3504	97.3	79.2	-163.4
42	4.3603	88.9	-177.9	-66.8
43	4.4019	-147.8	-46.8	65.8

44	4.4057	79.7	155.4	-88.8
45	4.4064	-155.4	133.3	-110.9
46	4.4222	-155.3	-40.4	72.5
47	4.4891	-144	140.6	-103.7
48	4.5187	65.6	70.4	-173.6
49	4.5397	-142.1	-57.2	55.4
50	4.5454	86.5	73.1	-170
51	4.5741	87.6	77.7	-165.2
52	4.6065	76.1	150.5	-92.9
53	4.6174	96.5	79.5	-163
54	4.6227	-153.4	-43.3	69.6
55	4.6687	-127.4	-165.8	-55.6
56	4.6741	78.1	155	-89.1
57	4.71	-143.4	-56.9	55.8
58	4.7423	-133.4	-163.8	-52.9
59	4.7507	-154.4	-42	71
60	4.7661	-134.6	-165.6	-54.8
61	4.8225	74.6	150.3	-93
62	4.9011	85.8	73.6	-169.5
63	4.9217	65.7	68.7	-175.5
64	4.9465	-155.1	132.4	-111.3
65	5.0236	-155.8	-43.7	69.1
66	5.0359	-143	-56.8	55.9
67	5.0435	101.8	118.4	-128.4
68	5.061	-144.2	144.5	-100
69	5.0817	102.3	118.4	124.8
70	5.0985	-141.8	-57.4	55.2
71	5.1677	-125.6	-164.4	-54.1
72	5.2277	81.8	-57	55.9
73	5.2449	71.2	74.9	-168
74	5.2477	82.4	-56.7	56.1
75	5.2605	99.5	118.5	-125.2
76	5.2958	100.2	118.6	-125.1
77	5.3744	93.8	93.3	-148.8
78	5.3913	96.4	77.1	-165.5
79	5.4288	-151.2	134.1	-108.2
80	5.4354	-158.2	137.4	-106.9
81	5.4552	-155.5	134.8	-109.1
82	5.4756	92.9	112.8	-130.8
83	5.488	77.2	87.9	-154
84	5.4976	-132.5	-164.5	-53.6
85	5.5055	-133.8	-165.9	-55.1
86	5.5131	71.3	75.4	-167.3
87	5.5553	-155.7	133.5	-110.6
88	5.5801	94.6	92.8	-149.3
89	5.5916	94.7	-67.5	45.1
90	5.5973	74.1	80	-161.6
91	5.6104	71.1	84.6	-158
92	5.6428	98.2	-172.4	-61.5

93	5.6484	95.5	77.4	-165.2
94	5.6662	93.9	111.9	-131.5
95	5.7065	77.4	88.3	-153.4
96	5.7741	74.4	80.6	-160.9
97	5.8439	75	62.9	177.5
98	5.8592	101	103.8	-139.1
99	5.8785	70.2	85.1	-157.6
100	6.0457	101.1	103.6	-139.3

[a.] Conformers showing dihedral angles over 140° were selected and the vinyl $^{3}J_{H,H}$ values can be estimated using the modified Karplus Equation by Garbisch (Garbisch, E. W., Jr. *J. Am. Chem. Soc.* **1964**, *86*, 5561–5564). [b.] Small $^{3}J_{H,H}$ (< 4 Hz) values were selected based on dihedral angles in the range 50–120° and large $^{3}J_{H,H}$ (> 8 Hz) values were in the ranges 0–30° and /or 140–180°.

Table S8. Conformers of 1*R**,2*R**,7*S**,8*R**-**2** for *J*-DP4 calculation and the dihedral angles of selected protons at MMFF94 level (the selected conformers were marked in yellow).

	MMFF94 rel. energy	Dihedral angles at MMFF94 level		
**conformer**	**(kcal/mol)**	**H-2/H-3**[a]	**H-7/H-6 *pro-R***[b]	**H-7/H-6 *pro-S***[b]
1	0	-147.9	81.3	-166.1
2	1.4734	-145.2	79.9	-167.9
3	1.5167	-146.4	90.8	-156.3
4	1.8714	-148.1	81.7	-165.6
5	2.6284	-147.4	-89	24.5
6	2.7953	-138	147.4	-95.7
7	3.3378	-142	66.1	177.3
8	3.356	-111.9	173.9	-68.5
9	3.4379	-145.5	77.8	-170
10	3.4861	-143.4	66.6	178
11	3.5591	-141.7	64.7	175.6
12	3.8114	-143.1	65.1	176.2
13	3.8997	-141.1	115.6	-128.2
14	4.1878	-112	174.5	-68
15	4.2191	-156.7	-93.8	19.4
16	4.2554	-144.7	144.7	-98.3
17	4.3661	-147.6	121.6	-121.7
18	4.3748	-143.7	-86.7	26.6
19	4.7025	-156.2	-96.2	17.1
20	4.7456	-153.1	86.3	-160.2
21	4.828	-150.4	147.1	-95.8
22	5.0198	-143.6	-86.9	26.7
23	5.037	-133.9	155.9	-86.8
24	5.0635	-152.5	83.8	-162.8
25	5.4057	-148.4	85.4	-160.4
26	5.454	-149.2	-87.2	26.3
27	5.4741	-153	79.4	-167.9
28	5.521	-151.7	146	-96.7
29	5.5485	-141.7	64.7	175.7
30	5.6026	-144.7	145.4	-98
31	5.6295	-143	65.2	176.4
32	5.6586	-156.1	-91.5	21.7
33	5.6598	-141.4	63.5	174.2
34	5.7175	-150.1	148.2	-94.8
35	5.8477	-142.8	63.8	174.7
36	5.8509	-141.3	64.8	175.8
37	5.9047	-148.8	-88.1	25.4
38	5.9216	-142.6	65.2	176.4
39	5.9331	-141	63.6	174.2
40	5.9465	-106.9	171.4	-70.8
41	6.0556	-132.9	159.1	-83.6

[a.] Conformers showing dihedral angles over 140° were selected and the vinyl $^3J_{H,H}$ values can be estimated using the modified Karplus Equation by Garbisch (Garbisch, E. W., Jr. *J. Am. Chem. Soc.*

1964, *86*, 5561–5564). [b] Small $^3J_{H,H}$ (< 4 Hz) values were selected based on dihedral angles in the range 50–120° and large $^3J_{H,H}$ (> 8 Hz) values were in the ranges 0–30° and /or 140–180°.

Table S9. Conformers of 1*R**,2*R**,7*R**,8*S**-**2** for *J*-DP4 calculation and the dihedral angles of selected protons at MMFF94 level (the selected conformers were marked in yellow).

	**MMFF94 rel. energy**	**Dihedral angles at MMFF94 level**		
**conformer**	**(kcal/mol)**	**H-2/H-3**[a]	**H-7/H-6 *pro-R***[b]	**H-7/H-6 *pro-S***[b]
1	0.000	-147.6	-178.8	-66.8
2	0.204	-150.3	-177.7	-65.7
3	1.726	-138.3	65.3	-177.4
4	2.220	-145.2	178.7	-69.1
5	2.593	-148.6	-179.1	-66.9
6	4.093	-140.4	69	-173.6
7	4.444	-136.4	67.4	-174.9
8	4.795	-149.1	174.7	-71.5
9	4.968	-169.1	113.4	-130.2
10	5.042	-147.4	174.2	-72.1
11	5.2739	-150.1	-50.7	61.9
12	5.6045	-148.5	-50.8	61.8
13	5.871	-143.0	60.9	176.6

[a] Conformers showing dihedral angles over 140° were selected and the vinyl $^3J_{H,H}$ values can be estimated using the modified Karplus Equation by Garbisch (Garbisch, E. W., Jr. *J. Am. Chem. Soc.* **1964**, *86*, 5561–5564). [b] Small $^3J_{H,H}$ (< 4 Hz) values were selected based on dihedral angles in the range 50–120° and large $^3J_{H,H}$ (> 8 Hz) values were in the ranges 0–30° and /or 140–180°.

Table S10. Conformers of 1*R**,2*R**,7S*,8S*-**2** for *J*-DP4 calculation and the dihedral angles of selected protons at MMFF94 level (none of the conformers was selected).

	**MMFF94 rel. energy**	**Dihedral angles at MMFF94 level**		
**conformer**	**(kcal/mol)**	**H-2/H-3**[a]	**H-7/H-6 *pro-R***[b]	**H-7/H-6 *pro-S***[b]
1	0.000	72	103.8	-140.8
2	0.137	87.5	-72.9	39.4
3	0.442	75.6	122.9	-121.1
4	0.513	82.9	106.5	-138.1
5	0.664	71.1	-73.6	38.6
6	0.772	71.5	101	-143.8
7	0.916	69.8	105.5	-139.1
8	1.175	75	103.7	-140.9
9	1.290	73.5	109.8	-134.7
10	1.601	-147.5	-73.2	38.9
11	1.685	-147.1	103.2	-141.5
12	1.969	76.5	108.8	-135.5
13	2.022	-148.3	-74.3	37.7
14	2.052	87.1	125.5	-118.7
15	2.428	91.5	124.3	-119.5
16	2.461	-144.5	55.6	166.1
17	2.517	93.4	123.8	-120.1
18	2.553	-148.3	155.7	-86.8
19	2.858	71.5	53.4	164.1
20	2.996	72.3	56.1	166.6
21	2.997	91.8	111.9	-132.9
22	3.282	70.6	56.1	166.4
23	3.284	-144.4	114	-130.8
24	3.344	99.1	-176.6	-60.2
25	3.413	99.7	54	164.8
26	3.484	91.3	110.1	-134.7
27	3.643	92.9	143	-99.5
28	3.660	92.2	-174.2	-58.3
29	3.893	-157.5	105	-139.7
30	3.938	93.9	113	-131.5
31	3.961	88.2	111.7	-133.1
32	4.039	-134.1	-79.6	32.9
33	4.081	-135.3	127.5	-116.6
34	4.081	73.3	99.8	-145
35	4.095	97.3	111.2	-133.1
36	4.115	88.9	152.5	-89.5
37	4.142	79.7	126.6	-117.6
38	4.154	-155.4	54.8	165.2
39	4.165	-144	143.6	-99
40	4.254	65.6	56.6	166.9
41	4.490	86.5	114.1	-130.2
42	4.586	87.6	-177.3	-60.8
43	4.590	76.1	155.7	-86.7
44	4.593	96.5	-77.1	35.5

45	4.619	-127.4	141.6	-100.8
46	4.624	78.1	113	-129.5
47	4.627	-133.4	155.6	-86.9
48	5.108	-134.6	-171.1	-55
49	5.1161	73	55.6	166
50	5.1945	74.6	142	-100.4
51	5.3231	94.2	117.2	-125.2
52	5.3364	90.3	-174.4	-57.9
53	5.406	90	-176.3	-59.8
54	5.6019	101	-73.5	39.5
55	5.6615	69.7	-174.1	-58.3
56	5.6656	87.6	59.3	171.3
57	5.692	68.5	-72.1	40.1
58	5.786	73.3	-65.4	48.9
59	5.8481	72.7	-64.9	49.2
60	5.8582	99	-72.1	41.1
61	5.9031	70.4	-66.7	45.7
62	5.9453	69.4	44.6	-67.8

[a.] Conformers showing dihedral angles over 140° were selected and the vinyl $^3J_{H,H}$ values can be estimated using the modified Karplus Equation by Garbisch (Garbisch, E. W., Jr. *J. Am. Chem. Soc.* **1964**, *86*, 5561–5564). [b.] Small $^3J_{H,H}$ (< 4 Hz) values were selected based on dihedral angles in the range 50–120° and large $^3J_{H,H}$ (> 8 Hz) values were in the ranges 0–30° and /or 140–180°.
